# A newly recognized theropod assemblage from the Lewisville Formation (Woodbine Group; Cenomanian) and its implications for understanding Late Cretaceous Appalachian terrestrial ecosystems

**DOI:** 10.7717/peerj.12782

**Published:** 2022-01-25

**Authors:** Christopher R. Noto, Domenic C. D’Amore, Stephanie K. Drumheller, Thomas L. Adams

**Affiliations:** 1 Department of Biological Sciences, University of Wisconsin-Parkside, Kenosha, Wisconsin, United States; 2 Department of Natural Sciences, Daemen College, Amherst, New York, United States; 3 Department of Earth and Planetary Sciences, University of Tennessee, Knoxville, Tennessee, United States; 4 Witte Museum, San Antonio, Texas, United States

**Keywords:** Cenomanian, Theropoda, Appalachia, Transition, Mid-Cretaceous, Tyrannosauroidea, Ornithomimosauria, Troodontidae, Dromaeosauridae, Carcharodontosauria

## Abstract

While the terrestrial fossil record of the mid-Cretaceous interval (Aptian to Cenomanian) in North America has been poorly studied, the recent focus on fossil localities from the western United States has offered a more detailed picture of vertebrate diversity, ecosystem dynamics and faunal turnover that took place on the western landmass of Laramidia. This is in stark contrast to the terrestrial record from the eastern landmass of Appalachia, where vertebrate fossils are rare and consist mostly of isolated and fragmentary remains. However, a detailed understanding of these fossil communities during this interval is necessary for comparison of the faunal patterns that developed during the opening of the Western Interior Seaway (WIS). The Woodbine Group of Texas is a Cenomanian age (95–100 mya) deposit consisting of shallow marine, deltaic, and terrestrial communities, which were only recently separated from their western counterparts. These deposits have yielded a wealth of vertebrate remains, yet non-avian theropods are still largely unknown. Recently, multiple localities in the Lewisville Formation of the Woodbine Group have yielded new non-avian theropod material, including numerous isolated teeth and postcranial remains. While largely fragmentary, this material is sufficiently diagnostic to identify the following taxa: a large-bodied carcharodontosaur, a mid-sized tyrannosauroid, a large ornithomimosaur, a large dromaeosaurine, a small dromaeosaurid, a small troodontid, and a small coelurosaur. Some of these groups represent the first occurrence for Appalachia and provide a broader understanding of a newly expanded faunal diversity for the Eastern landmass. The Lewisville Formation theropod fauna is similar in taxonomic composition to contemporaneous deposits in Laramidia, confirming that these groups were widespread across the continent prior to extension of the WIS. The Lewisville Formation documents the transitional nature of Cenomanian coastal ecosystems in Texas while providing additional details on the evolution of Appalachian communities shortly after WIS extension.

## Introduction

The mid-Cretaceous (approximately the Aptian to Cenomanian) is a time period of major turnover in terrestrial ecosystems, when taxa that would become dominant components of Late Cretaceous communities and the “modern” terrestrial fauna first appear (Benson et al., 2013; Jacobs & Winkler, 1998; Nesbitt et al., 2019; Pérez-García et al., 2020; Zanno & Makovicky, 2013). Yet globally, and particularly in North America, there remains a paucity of fossil data from the mid-Cretaceous, where the record of non-avian theropods is poorly known and consists primarily of fragmentary skeletal remains and teeth. The most diverse theropod assemblage from this interval occurs in the Mussentuchit Member of the Cedar Mountain Formation, including remains of the carcharodontosaur *Siats meekerorum*, small tyrannosauroids, tyrannosaurids, troodontids, dromaeosaurids, and an oviraptorosaur (Kirkland & Madsen, 2007; Zanno & Makovicky, 2011; Zanno & Makovicky, 2013). Rare tooth remains of dromaeosaurids, troodontids, cf. *Richardoestesia*, and a tyrannosaurid are known from the Naturita (“Dakota”) Formation of the Kaiparowits Plateau (Eaton et al., 1999). Fragmentary remains of a tyrannosauroid and dromaeosaurid are known from the Blackleaf Formation of Montana (Ullmann, Varricchio & Knell, 2012). Recently described theropod remains from the Wayan Formation of Idaho consist of tyrannosauroids, dromaeosaurids, a possible neovenatorid, and eggshell of the oviraptorosaur ootaxon *Macroelongatoolithus* (Krumenacker et al., 2016).

However, this record only provides half of the picture. Beginning in the Albian, North America was gradually divided into two separate landmasses by the incursion of the Western Interior Seaway (WIS), forming the landmasses of Laramidia to the west and Appalachia to the east; a condition that persisted until at least the Maastrichtian (Slattery et al., 2015). Thus, the history of North America for the majority of the Late Cretaceous is divided between Laramidia and Appalachia. To date this record has been almost entirely Laramidian in origin, providing crucial insight into the timing and tempo of changes in North American terrestrial ecosystems, particularly in the large-bodied predator guild inhabited by various theropod groups (Zanno & Makovicky, 2011; Zanno & Makovicky, 2013; Zanno et al., 2019). In contrast, the mid-Cretaceous non-avian theropod record from Appalachia is exceedingly sparse, consisting only of a set of isolated teeth from the Lewisville Formation in Texas referred to *Richardoestesia* (Lee, 1997a) and a possible ornithomimid from the McShan/Eutaw Formation of Mississippi (Carpenter, 1982). This sparse record throughout the mid-Cretaceous interval severely limits understanding of biogeographic and evolutionary patterns on this landmass (Carr, Williamson & Schwimmer, 2005). This knowledge gap becomes critical when interpreting records of non-avian theropods in Campanian-Maastrichtian deposits of the eastern United States, which has led to a variety of interpretations including the possibility of endemic or relict assemblages (Brownstein, 2017b, 2018b, 2018c, 2019; Carr, Williamson & Schwimmer, 2005; Kiernan & Schwimmer, 2004; Schwimmer et al., 2015; Schwimmer et al., 1993).

Ongoing work in the Lewisville Formation exposures around the Dallas–Fort Worth area of north central Texas helps address this gap in the Appalachian non-avian theropod record. Investigations of the Lewisville Formation vertebrate fauna stretch back decades, including fish (Main et al., 2011; McNulty & Slaughter, 1962; McNulty & Slaughter, 1968), mammals (Krause & Baird, 1979), turtles (Adrian et al., 2019; Adrian et al., 2021), crocodyliforms (Adams, 2013; Adams, Noto & Drumheller, 2017; Adams et al., 2011; Lee, 1997a; Noto et al., 2020), and dinosaurs (Head, 1998; Jacobs & Winkler, 1998; Lee, 1997a; Main, Noto & Weishampel, 2014; Winkler et al., 1995; Winkler & Jacobs, 2002). Much of the vertebrate material recovered from the Lewisville Formation is isolated and fragmentary, making comprehensive study of the remains and their affinities difficult (Drumheller et al., 2021). This paper describes isolated non-avian theropod teeth and postcranial material recovered from multiple localities. This material includes specimens recovered by the authors, as well as previously recovered specimens identified in museum collections. These specimens add significantly to the known non-avian theropod diversity of Appalachia in the Cenomanian, representing an important comparison to the better-known assemblages from the west, and provide critical context for understanding the fauna in the east that followed during the Campanian and Maastrichtian.

## Age and geologic setting

The terminology and understanding of the Woodbine Group is complex, with differing interpretations and nonmenclature based on studies of surface exposures *vs* subsurface drill cores and wireline logs, as well as a long history of revisions in stratigraphic subdivision (Ambrose et al., 2009; Bergquist, 1949; Dodge, 1952; Dodge, 1968; Hentz, Ambrose & Smith, 2014; Johnson, 1974; Murlin, 1975; Oliver, 1971; Trudel, 1994). The Woodbine Group is the oldest Upper Cretaceous unit on the Gulf Coastal Plain (Hedlund, 1966; Oliver, 1971), classified as a third order regressive sequence deposited over ~1.5 million years (Ambrose et al., 2009). On the surface the Woodbine Group is exposed in a narrow, irregular band, stretching between Lake Texoma in southern Oklahoma southward to Temple in central Texas (Dodge, 1969; Johnson, 1974; Oliver, 1971; Trudel, 1994). In the study area it sits unconformably over the Grayson Marl (Washita Group) and is covered in another unconformity by the Eagle Ford Group. The Woodbine Group is separated from the older terrestrial units that distinguish the Lower Cretaceous Trinity Group by a period of marine deposition lasting at least ten million years (Winkler et al., 1995).

Stratigraphic subdivision of the Woodbine Group has undergone multiple changes, as the number and composition of subunits changes with location. Within the Woodbine Group, two units are currently recognized: the lower Dexter Formation representing marginal and marine environments (Bergquist, 1949; Dodge, 1952; Dodge, 1968; Dodge, 1969; Johnson, 1974; Oliver, 1971) and the overlying Lewisville Formation, which represents a low-lying coastal plain (Main, 2009; Oliver, 1971; Powell, 1968). Sequence stratigraphic and chronostratigraphic studies suggest the Woodbine Group is no older than middle-early Cenomanian (Adams & Carr, 2010; Ambrose et al., 2009; Donovan et al., 2015; Vallabhaneni et al., 2016). An age of early middle Cenomanian (approximately 96 Ma) is given for the Lewisville Formation and in the Tarrant Formation (lowermost Eagle Ford Group) based on the presence of the ammonite *Conlinoceras tarrantense* (a zonal marker for the base of the middle Cenomanian; Kennedy & Cobban, 1990; Emerson et al., 1994; Lee, 1997a; Jacobs & Winkler, 1998; Gradstein, Ogg & Smith, 2004). However, Ambrose et al. (2009) suggests the Lewisville Formation is as young as late Cenomanian, with overall deposition of the Woodbine Group ending around 92 Ma.

**Figure 1:**
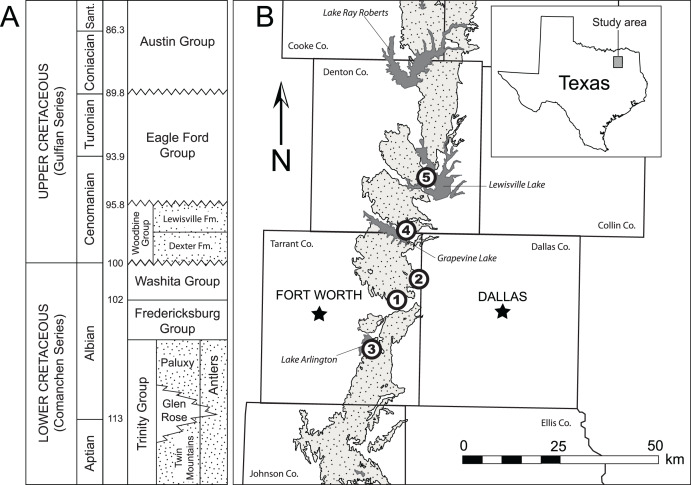
Location and geologic position of the Woodbine Group. (A) General stratigraphic sequence and timescale for the Cretaceous of central and north central Texas showing the position of the Woodbine Group. Terrestrial deposits represented by stippled intervals. Time scale based on Gradstein, Ogg & Smith (2004). Modified from Adams et al. (2011). B. Map of Woodbine surface exposures in the study area showing position of localities where fossils were discovered. Exposures are stippled, water bodies are solid gray. 1 = Arlington Archosaur Site, 2 = Bear Creek, 3 = Veteran’s Park, 4 = Grapevine Lake Spillway, 5 = Lewisville Lake.

The material described here was recovered from four different localities in Tarrant and Denton counties (Fig. 1). All four localities are placed within the middle to upper Lewisville Formation. Due to lack of surface exposures and disparate location of each site the precise stratigraphic relationship among the localities cannot be determined at present. A list of localities from which specimens were recovered is included in Table 1.

**Table 1:** Locality of discovery for each specimen described in this paper.

Specimen	Element	Taxon	Locality
DMNH 2013-07-0492	Phalanx	Theropoda	AAS
DMNH 2013-07-0494	Manual ungual	Carcharodontosauria	AAS
DMNH 2013-07-1080	Tooth	Dromaeosaurinae	AAS
DMNH 2013-07-1081	Tooth	Carcharodontosauria	AAS
DMNH 2013-07-1082	Tooth	Theropoda	AAS
DMNH 2013-07-1083	Tooth	Carcharodontosauria	AAS
DMNH 2013-07-1701	Tooth	Tyrannosauroidea	AAS
DMNH 2013-07-1990	Chevron	Carcharodontosauria	AAS
DMNH 2014-06-02	Tooth	Dromaeosaurinae	AAS
DMNH 2014-06-05	Tooth	Carcharodontosauria	Veteran’s Park
DMNH 2014-06-06	Tooth	Carcharodontosauria	Lake Lewisville
DMNH 18159	Manual ungual	Maniraptora	Grapevine Lake
SMU 73778	Tooth	Coelurosauria	SMU 245
SMU 73779	Tooth	Dromaeosauridae	SMU 245
SMU 76809	Tibia	Ornithomimosauria	SMU 245
SMU 76946	Tooth	Carcharodontosauria	SMU 245
SMU 76947	Tooth	Troodontidae	SMU 245
SMU 76948	Tooth	Dromaeosauridae	SMU 245
SMU 76949	Tooth	Carcharodontosauria	SMU 245
SMU 77213	Tooth	Coelurosauria	SMU 245
SMU 77214	Tooth	Coelurosauria	SMU 245
SMU 77217	Tooth	Dromaeosauridae	SMU 245
SMU 77218	Tooth	Tyrannosauroidea	SMU 245

### The Arlington Archosaur Site (AAS)

Tarrant County. AAS deposits represent a transition from freshwater or brackish wetland to near-shore marine environments. Exposures consist of an organic-rich shale (peat) dominated by carbonized plant matter, overlain by a gray mudstone-dominated paleosol with abundant charcoalified plant remains and calcareous nodules, then an oxidized coarse sand/pebble conglomerate, followed by interbedded fine sand and silty clay, capped with rippled sand beds. Numerous fossils have been described from here belonging to a variety of vertebrates, invertebrates, and plants (Adams, Noto & Drumheller, 2017; Adrian et al., 2019; Adrian et al., 2021; Main, 2013; Main, Noto & Weishampel, 2014; Main et al., 2011; Noto et al., 2020; Noto, Main & Drumheller, 2012). Specific locality coordinates are on file with the Perot Museum of Nature and Science.

### Bear Creek (SMU locality 245)

Tarrant County. Located near the south entrance to Dallas-Fort Worth International Airport. Exposures occur primarily along a cut bank of a large stream and mark a terrestrial to marine transition consisting of shaly sandstones, thin sandy layers interbedded with sandy shale, phosphatic lag deposits, and dark, carbonaceous strata that lie a few meters below the contact with the Eagle Ford Group (Lee, 1997a). Fossiliferous layers contain abundant reworked remains of vertebrates, primarily teeth, and found with fossils attributed to brackish environments. Larger skeletal elements, such as limb bones or vertebrae, were transported from unknown strata upstream and collected as surface float. Specific locality coordinates are on file with the Shuler Museum at Southern Methodist University.

### Veteran’s Park

Tarrant County. The majority of the bedding is obscured by topsoil and plants, but appears to represent a terrestrial environment composed of drab, gray-green mudstone, overlain by a pale, medium-grained sandstone, topped by a thin fossiliferous layer of medium-coarse, iron-rich sandstone that may represent terrestrial overbank deposits. Fossils here are rare and consist of isolated and/or fragmented elements collected as surface float. Specific locality coordinates are on file with the Perot Museum of Nature and Science.

### Grapevine Lake

Denton County. Extensive Woodbine exposures occur in the public lands surrounding Grapevine Lake, which is administered by the United States Army Corps of Engineers. Dinosaurs include isolated remains attributed to *Protohadros*, found in Murrell Park and nearby Rock Ledge Park (Main, 2005). Lee (1997b) described numerous dinosaur tracks exposed on the lake shore of Murrell Park, which he assigned to non-avian and avian theropods, and hadrosaurs. The material described here was collected as surface float at the Grapevine Lake Dam Spillway. Exposures around the spillway are typical of a coastal plain environment, consisting of interbedded fine sands and silts, interspersed with sandy channel and levee deposits, and abundant carbonized and petrified wood pieces, (Tykoski & Fiorillo, 2010). The enantiornithine bird *Flexomornis howei*, was discovered in this area (Tykoski & Fiorillo, 2010). Specific locality coordinates are on file with the Perot Museum of Nature and Science.

### Lake Lewisville

Denton County. The exposure contains repeating units of medium-coarse sandstone, finely laminated siltstone with ripple marks, and fossiliferous beds composed of iron-rich, coarse conglomeratic sand, which likely represents a near-shore marine environment proximal to an active fluvial system. The material was collected *in situ* while this site was exposed during an extraordinary drought in the area during 2013, which saw lake levels drop several meters. As of late 2015 the site is once again under water. Specific locality coordinates are on file with the Perot Museum of Nature and Science.

## Materials and Methods

Descriptive nomenclature for theropod teeth follow that established in Smith, Vann & Dodson (2005) and expanded by Hendrickx, Mateus & Araújo (2015). Specimens were examined with a Leica S6D zoom stereomicroscope, using an attached Leica EC3 digital camera to image each specimen. Due to the absence of contemporaneous, Appalachian assemblages upon which to base morphometric comparisons, and given the small sample size within the Lewisville Formation itself, we use a multifaceted method for clade-level identification of isolated crowns. First, we used previously recognized, apomorphic characters to determine the most inclusive clade to which the teeth could be assigned, based on recent phylogenetic analyses of major theropod groups (Averianov & Sues, 2019; Avrahami et al., 2018; Brusatte et al., 2010; Currie, Rigby & Sloan, 1990; Hendrickx et al., 2019; Longrich, 2008; Sankey et al., 2002; Turner, Makovicky & Norell, 2012; Williamson & Brusatte, 2014). Second, we conducted a morphometric analysis similar to Hendrickx, Tschopp & Ezcurra (2020). Measurements were taken from photographs of each tooth from multiple perspectives, including labial, mesial, and zoom-ins on the distal denticles, using the aforementioned camera. A scale was included in all photographs. Relevant photographs were then uploaded into the software TpsDig2.31 (Rohlf, 2018). Landmarks were plotted at the mesial and distal base at the enamel margin, and the apical most point on photos from the labial perspective. Connecting these points resulted in the Crown Base Length (CBL), Crown Height (CH), and Apical Length (AL). Landmarks were also plotted midway up the CH on the mesial and distal margins, and the distance between the two resulted in the Mid-crown Length (MCL). In photographs from the mesial perspective, landmarks were plotted at the enamel margin and midway up the crown to derive Crown Base Width (CBW) and Mid-crown Length (MCW) (found in Table 2). For denticle measurements, we took the Distal Denticle Length (DDL) of a single distal denticle midway up the length of the crown and Mesial Denticle Length (MDL) of a single distal denticle two-thirds up the length of the crown (Table 3). These were derived by plotting landmarks at the ampulla on either side of the designated denticle and the base of the interdenticular diaphysis. Denticle Size Density Index (DSDI) is the ratio of MDL to DDL (modified from Smith et al., 2005, Table 2). We also recorded the presence and number of flutes along the labial and lingual surfaces (LAF and LIF respectively). From these measurements we extrapolated Crown-Base Ratio (CBR), Crown-Height Ratio (CHR), Mid-crown Ratio (MCR), and Crown Angle (CA) (Table 2). For a complete description of these measurements, see Hendrickx, Mateus & Araújo (2015).

**Table 2:** Selected morphometric measurements for theropod tooth specimens.

Specimen	Taxon	CBL	CBW	CH	AL	CBR	CHR	DSDI	Enamel texture
DMNH 2013-07-1080	Dromaeosaurinae	12.13	6.16	(16.46)	(20.96)	0.51	(1.36)	1.14	braided
DMNH 2013-07-1081	Carcharodontosauria	(17.29)	8.40	(44.79)	(46.69)	(0.49)	(2.59)	1.10	braided
DMNH 2013-07-1082	Theropoda	4.90	2.60	10.60	12.00	0.53	2.16	1.44	uncertain
DMNH 2013-07-1083	Carcharodontosauria	7.18	3.56	(17.54)	(17.47)	0.50	(2.44)	1.04	braided
DMNH 2013-07-1701	Tyrannosauroidea	(13.02)	(8.29)	(21.32)	24.56	(0.64)	(1.64)	1.72	irregular/smooth
DMNH 2014-06-02	Dromaeosaurinae	8.72	4.32	13.93	15.88	0.49	1.60	1.11	braided
DMNH 2014-06-05	Carcharodontosauria							1.32	braided
DMNH 2014-06-06	Carcharodontosauria	16.05	7.99	(37.30)	(40.26)	0.50	(2.32)	1.07	braided
SMU 73778	Coelurosauria indet.	1.30	0.73	1.98	2.07	0.56	1.52	0.93	irregular/smooth
SMU 73779	Dromaeosauridae	2.31	0.93	3.22	3.60	0.40	1.40		braided
SMU 76946	Carcharodontosauria	13.72	5.97	(37.00)		0.43	2.70	1.24	braided
SMU 76947	Troodontidae	3.00	1.70	5.83	6.57	0.57	1.94	0	irregular/smooth
SMU 76948	Dromaeosauridae	2.21	0.97	3.26	3.57	0.44	1.48		braided
SMU 76949	Carcharodontosauria								braided
SMU 77213	Coelurosauria		1.30					0.97	irregular/smooth
SMU 77214	Coelurosauria								irregular/smooth
SMU 77217	Dromaeosauridae	3.40	1.30	(5.00)		0.38	(1.50)	1.13	braided
SMU 77218	Tyrannosauroidea	(7.02)	4.07	(9.15)	10.51	(0.58)	(1.30)	1.05	irregular/smooth

**Note:**

CBL, Crown Base Length; CBW, Crown Base Width; CH, Crown Height; AL, Apical Length; CBR, Crown-Base Ratio; CHR, Crown-Height Ratio; and DSDI, denticle size density index. Enamel texture is based on Hendrickx et al. (2019).

**Table 3:** Body length estimates for theropod teeth based on Mesial and Distal Denticle Lengths (MDL and DDL respectively), and calculated using the results of D’Amore & Blumenschine (2012).

Taxon	Specimen	Denticle height (mm)		Body length estimate (m)	
		Mesial denticles	Distal denticles	Mesial denticles	Distal denticles
Carcharodontosauria	DMNH 2013-07-1081	0.322	0.356	4.6	5.7
	DMNH 2013-07-1083	0.233	0.261	3.0	3.2
	DMNH 2014-06-05	na	0.310	na	4.3
	DMNH 2014-06-06	na	0.329	na	4.9
	SMU 76946	0.252	0.331	3.2	4.9
	SMU 76949	na	0.320	na	4.6
Tyrannosauroidea	DMNH 2013-07-1701	na	0.327	na	4.8
	SMU 77218	0.215	0.253	2.7	3.1
Dromaeosaurinae	DMNH 2013-07-1080	0.349	0.336	5.3	5.1
	DMNH 2014-06-02	na	0.283	na	3.6
Dromaeosauridae	SMU 73779	0.093	0.124	1.5	1.4
	SMU 76948	na	0.151	na	1.6
	SMU 77217	0.125	0.175	1.7	1.9
Troodontidae	SMU 76947	na	0.348	na	5.5*
Coelurosauria	SMU 73778	0.106	0.101	1.6	1.2
	SMU 77213	0.112	0.147	1.6	1.6
	SMU 77214	0.085	na	1.4	na
Theropoda	DMNH 2013-07-1082	0.207	0.223	2.5	2.6

**Note:**

Estimates based on apical-basal lengths of both mesial and distal denticles were calculated, and used to find body length for each taxon. Specimens where denticles were lacking are indicated with ‘na’. Because troodontid denticles are abnormally large compared to other theropods of comparable size (D’Amore & Blumenschine, 2012) these length estimates are not used, as indicated by an asterisk (*).

The quality of the preserved dental material was highly variable (see below). We therefore only collected data from 12 of the isolated teeth in our data set. Of these 12, not all the landmarks could be plotted with confidence. We subsequently compiled two data sets. The former only consists of measurements taken from landmarks plotted on clear anatomical structures discussed above, referred to from here on as ‘observed’ data. The second also includes ‘reconstructed’ data, which are based on estimations of where the landmarks would be plotted if the teeth were complete. The most commonly reconstructed measurements involved estimating the position of missing corners of damaged teeth. Often the base was chipped on the mesial and/or distal side. For these we extended the enamel margin to where we believed it would have ended. A similar method was used for broken apices, where the margins were continued to where the apex was believed to be. Missing denticles were not estimated, as we did not know if they were absent due to damage or because they were never present.

A large database of theropod tooth morphometric data has been accumulated for over 17 years in the published literature, and we used these data to categorize the Woodbine teeth reported on here. We used a modified iteration of the database included in supplemental information of Hendrickx, Tschopp & Ezcurra (2020), which itself was taken from numerous studies (including Buffetaut, Escuillié & Pohl, 2005; Gianechini et al., 2015; Hendrickx, Mateus & Araújo, 2015; Smith & Lamanna, 2006; Smith & Dalla Vecchia, 2006; Smith, Vann & Dodson, 2005; Young et al., 2019; Zanno et al., 2016). A total of 952 teeth from this set were used based on the fact that they all had at least CBL, CBW, CH, and AL data. The clades designated for all these teeth were taken from Hendrickx, Tschopp & Ezcurra (2020) as well, resulting in 19 monophyletic groups the Woodbine teeth could be grouped into. Our only adjustment was to remove three *Neovenator* crowns from the clade designated ‘Neovenatoridae’ and reclassified the remaining members as ‘Megaraptora.’ This was due to the controversial positioning of Megaratora within Avetheropoda, and whether or not it belongs within Neovenatoridae (Benson, Carrano & Brusatte, 2010; Porfiri et al., 2014; Delcourt & Grillo, 2018). We then performed a Discriminant Function Analysis (DFA), a commonly done multivariate procedure to achieve this (similar to Hendrickx, Tschopp & Ezcurra, 2020; Larson & Currie, 2013; Young et al., 2019) using PAST 4.07b and its LDA function (Hammer, Harper & Ryan, 2001). Our unknowns were combined with the 955 teeth and all morphometric data available (except DSDI) was log^10^ scaled and used in the analysis. Two analyses were run: one on the reconstructed data, and one with only the directly observed measurements. Principal Component output was used to plot the unknown teeth against the published dataset for both groups of measurements. (See Supplemental Information for statistical data and analyses).

To facilitate comparisons of body size among the taxa recovered, an estimate of body length based on average denticle width was calculated using the results of D’Amore & Blumenschine (2012).

**Systematic paleontology and specimen descriptions**

TETANURAE Gauthier, 1986

ALLOSAUROIDEA Marsh, 1878

CARCHARODONTOSAURIA Benson, Carrano & Brusatte, 2010

Referred material–DMNH 2013-07-0494, DMNH 2013-07-1081, DMNH 2013-07-1083, DMNH 2013-07-1255, DMNH 2013-07-1990, DMNH 2014-06-05, DMNH 2014-06-06, SMU 76946

***Description:***

**DMNH 2013-07-1081, DMNH 2013-07-1083, DMNH 2013-07-1255, DMNH 2014-06-05, DMNH 2014-06-06 and SMU 76946**

**Figure 2:**
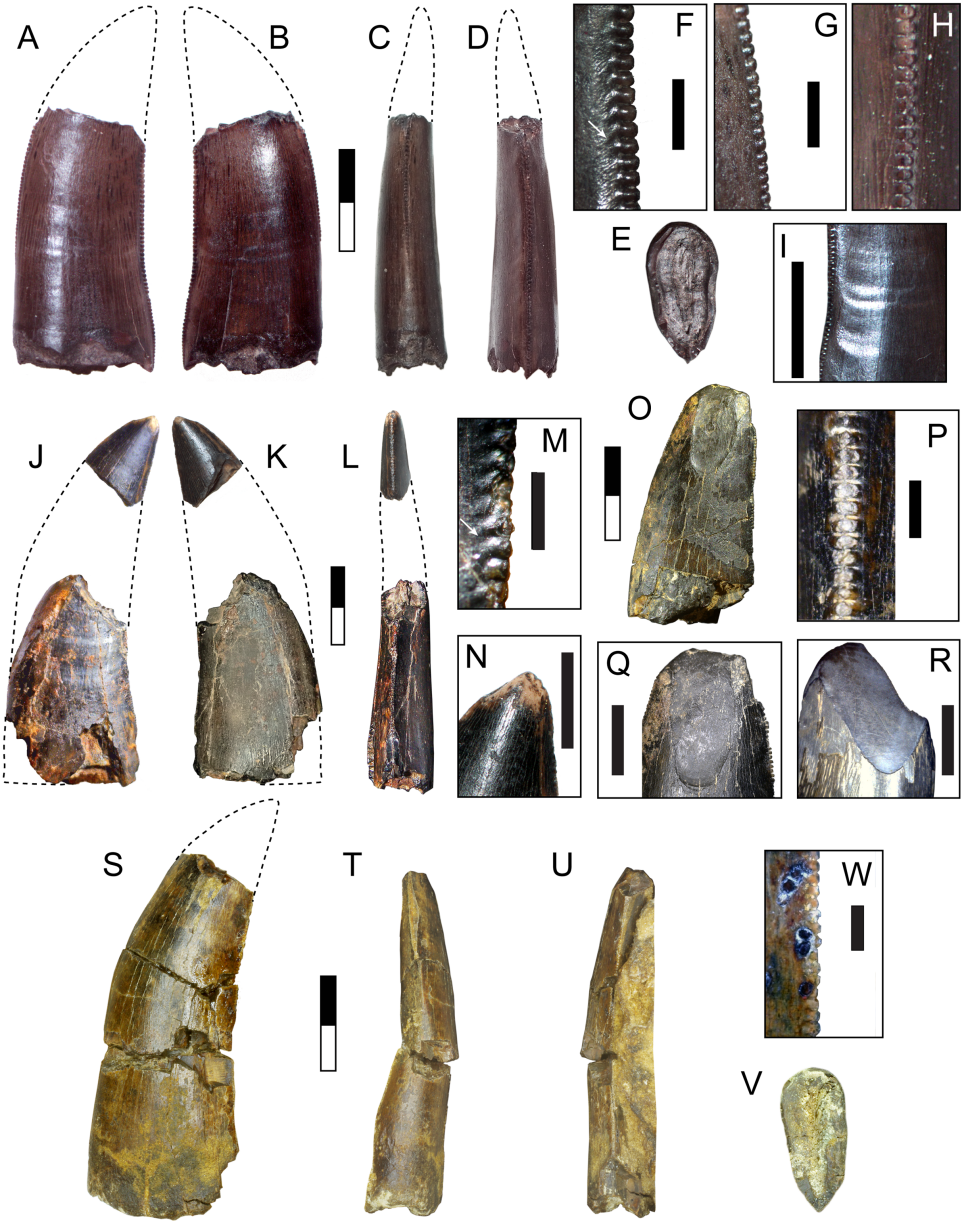
Teeth assigned to Carcharodontosauria. DMNH 2013-07-1083 showing lingual (A), labial (B), mesial (C), distal (D), basal (E) views and distal denticles (F), mesial denticles (G), distal denticles in posterior view (H), and enamel undulations (I). DMNH 2013-07-1081, showing labial (J), lingual (K), distal (L) views, and distal denticles (M), and enamel spalled surface (N). SMU 76946 showing lingual (O) view, posterior view of distal denticles (P), and enamel spalled surface (Q). (R) DMNH 2013-07-1255 showing enamel spalled surface. DMNH 2014-06-06 showing lingual (S), mesial (T), distal (U), and basal (V) views and distal denticles (W). White arrows indicate basally-oriented interdenticular sulci. Scale bars of unbordered images in A–E are 5 mm, (J)–(V) are 10 mm. Scale bars of bordered images are 1 mm.

Complete specimens of this tooth morphotype are lacking, however enough overlap between partial specimens exists to provide a reasonably complete picture of its morphology (Fig. 2; Table 1). This tooth morphotype has a laterally compressed, slightly flattened oval base and is moderately recurved. DMNH 2013-07-1083 possesses shallow depressions on both labial and lingual sides of the base. The teeth are laterally compressed (average CBR = 0.48: Table 2). They also appear to have a highly elongate crown and narrow base, which was apparent even though the apices were typically broken (average CHR = 2.58: Table 2). The mesial and distal margins run nearly parallel until mid-crown, and then have a sharp posterior deflection. The margins are therefore relatively straight, giving the teeth a distinctive rectilinear profile despite being recurved. The mesial carina extends one-half to three-quarters the distance to the cervix, while the distal carina extends basally beyond the cervix. Both carinae have a gentle labial, then lingual S-shaped curve, following the contours of the tooth. A majority of the mesial denticles are damaged. Those that are preserved are small (0.23–0.32 mm: Table 3) and short with a rounded profile in lateral view, becoming mammillate near the apex. The distal denticles trend larger (0.26–0.36 mm: Table 3) and subquadrangular in shape with symmetrical subrectangular to rounded external margins. The interdenticular space is shallow and the interdenticular slit between adjacent denticles is wide. Short, basally inclined interdenticular sulci are present on the distal margin of all teeth. Transverse enamel undulations are visible on both labial and lingual surfaces. These are composed of repeating groups of 2–3 prominent undulations separated by wider bands of smooth or weakly wrinkled enamel. The enamel surface texture is braided. Three specimens (DMNH 2013-07-1081, DMNH 2013-07-1255, SMU 76946) demonstrate enamel spalling, creating a flat or convex, concoidal-shaped surface on the apical end. Some teeth exhibit shallow, heterogeneously oriented scratches on the spalled surfaces that may be taphonomic in origin (Figs. 2C, 2D).

**DMNH 2013-07-0494**

**Figure 3:**
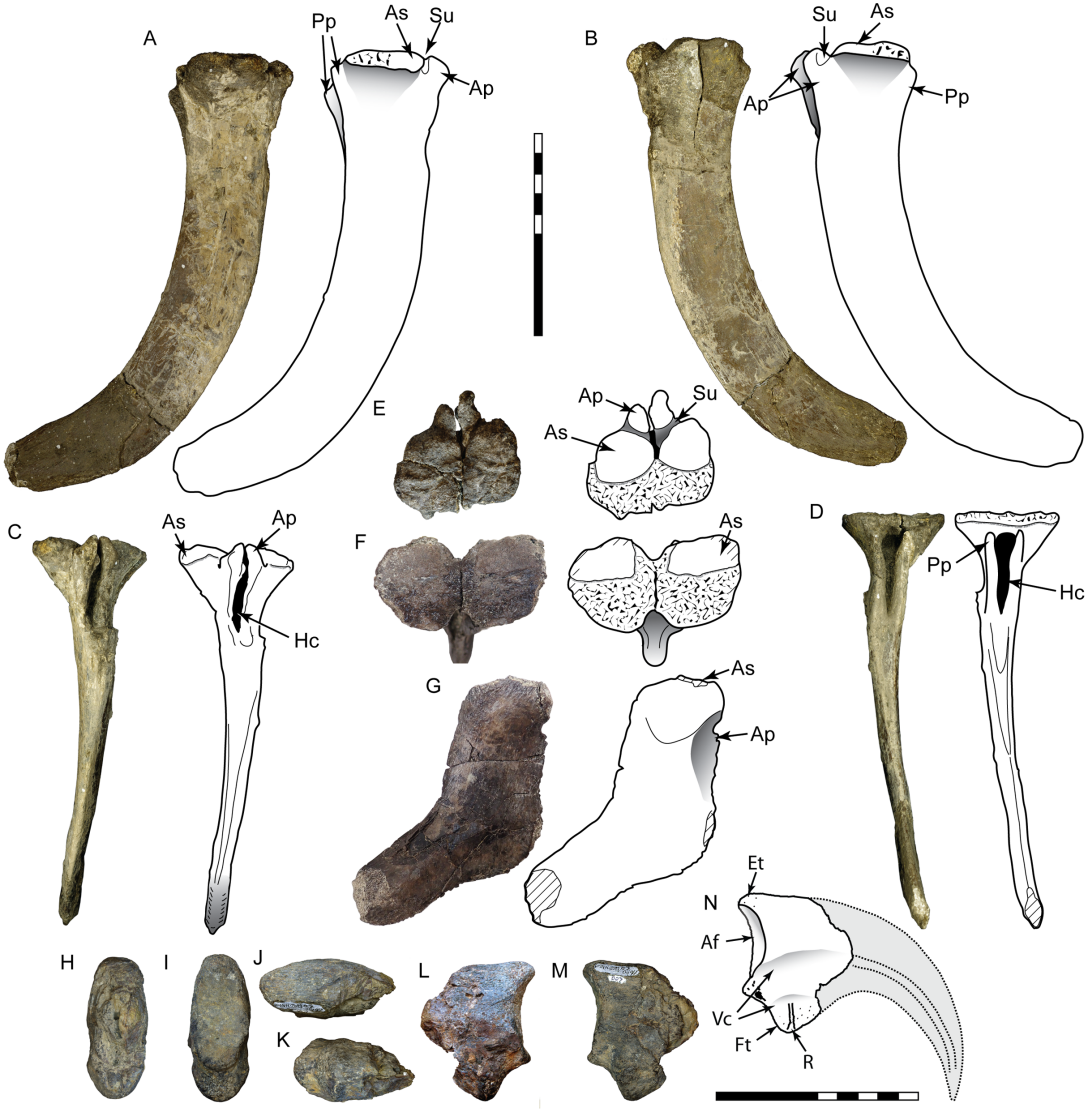
Postcranial material assigned to Carcharodontosauria. Chevron DMNH 2013-07-1990 in right lateral (A), left lateral (B), anterior (C), posterior (D), and proximal (E) views. FMNH PR 2716, chevron of *Siats meekerorum* (Zanno & Makovicky, 2013) in proximal (F) and right lateral (G) views. Manual ungual DMNH 2013-07-0494 in distal (H), proximal (I), dorsal (J), ventral (K), and lateral (L–M) views; reconstruction of complete element in lateral view (N) with reconstruction of missing portion (dashed line filled with grey) based on *Suchomimus* (Sereno et al., 1998). Ap, anterior process; As, articular surface; Af, articular facet; Ed, attachment for extensor tendon; Ft, flexor tubercle; Hc, haemal canal; Pp, posterior process; R, ridge; Su, sulcus; Vc, neurovascular canal. All scale bars are 10 cm.

This incomplete manual ungual is lacking the distal end and the distal portion of the flexor tubercle (Fig. 3). The overall length and curvature is uncertain, though it was likely recurved as in most theropods. The preserved proximal portion is mediolaterally compressed and measures 65 mm in total height. The articular facet is oval in proximal view with a height of 42 mm and width of 28 mm. The extensor tubercle is rounded and slightly dorsally everted, grading ventrally into a low median ridge on the surface of the articular facet. The flexor tubercle forms a pendulous, rounded point and measures about 20 mm in height. In lateral view the distal portion of the flexor tubercle slopes gently to meet the ventral surface of the ungual body, while the proximal surface is concave, creating a distally inset indentation from the ventral rim of the articular facet. In posterior view the flexor tubercle is constricted dorsally for passage of vascular canals. A distinct ridge traverses the lateral and medial sides, separating the body of the flexor tubercle into proximal and distal parts. The symmetrical surfaces of the articular facet suggest that this is phalanx II-3 (White et al., 2012).

**DMNH 2013-07-1990**

This is a complete, well-preserved chevron with a maximum length of 230 mm (Fig. 3). The haemal canal is enclosed proximally by a flat, rectangular shelf of bone 37 mm long and 56 mm wide that forms the articular surface with the caudal vertebrae. The anterior half of this rectangular articular surface contains two rounded facets that are slightly raised relative to the rest of the surface. Despite minor crushing on the proximal end, the haemal canal appears keyhole-shaped, approximately 12 mm wide and 42 mm tall, flanked laterally by transversely thin rami. The anterior surface of the rami possess a pair of anterodorsally-projecting laminae that terminate in enlarged, rectangular processes. These processes are closely-spaced and separated from the articular surface dorsally by a pronounced sulcus. A smaller pair of posterior processes is present on the rami, which grade into narrow laminae distally. The shaft is distinctly curved posteriorly on both anterior and posterior edges and transversely compressed. The distal end is rounded and unexpanded.

***Comparisons:***

These isolated specimens, found in multiple localities, are all identifiable as carcharodontosaurs based on numerous apomorphies. The teeth possess many characters found in the Carcharodontosauria, including transverse enamel undulations, basally inclined interdenticular sulci, and S-shaped mesiodistal profile (Benson, Carrano & Brusatte, 2010; Coria & Currie, 2006; Currie & Azuma, 2006; Currie & Carpenter, 2000; Harris, 1998; Naish, 2011; Novas et al., 2013; Sereno et al., 1996). While tall and moderately recurved, this tooth morphotype lacks the extensive anterior carina, extreme labiolingual compression, and large size observed in carcharodontosaurids such as *Mapusaurus*, *Giganotosaurus*, and *Carcharodontosaurus* (Coria & Currie, 2006; Novas et al., 2013). It shares a rectilinear, moderately recurved shape and non-angled, rectangular (‘cartouche’) distal denticles with *Acrocanthosaurus*, but differs in its smaller size, narrower base, and presence of interdenticular sulci, transverse enamel banding, and apically-restricted mesial carina (Currie & Carpenter, 2000; Harris, 1998). This morphotype shares numerous characters with *Neovenator* including labiolingually narrow, highly elongate and mildly recurved crowns, low, rectangular denticles, and mesial denticles half the height of the distal denticles (Brusatte, Benson & Hutt, 2008). Longitudinal depressions on labial and lingual surfaces are characters of Megaraptora, however this feature is found on only one specimen (DMNH 2013-07-1082) and is shallow and basally restricted, unlike the deeper and more apically extensive depressions in megaraptorans (Novas, Ezcurra & Lecuona, 2008; Porfiri et al., 2014; White et al., 2015). This morphotype differs further in possessing interdenticular sulci and mesial denticles, both of which are absent in Megaraptora. The combined evidence supports a carcharodontosaurian identification for this morphotype. These specimens are comparable in size to many large tetanuran taxa, with an upper estimated body length of 5.7 m (Table 3).

The incomplete manual ungual (DMNH 2013-07-0494) is assigned to the Allosauroidea due to possessing the following characters: an oval-shaped articular facet, pendulous flexor tubercle with a dorsal constriction, and mediolateral compression (proximal height:width ratio of 2.32) (Benson, Carrano & Brusatte, 2010; Rauhut, 2003). DMNH 2013-07-0494 is similar to the unguals of carcharodontosaurids *Mapusaurus* and *Concavenator*, but differs markedly from the unguals of *Acrocanthosaurus*, which are proportionally smaller and less curved with a small, rounded flexor tubercle most likely related to specialized function of the forelimb during predation (Coria & Currie, 2006; Currie & Carpenter, 2000; Ortega, Escaso & Sanz, 2010; Senter & Robins, 2005). DMNH 2013-07-0494 shares similar dimensions to ungual II-3 of *Allosaurus*, yielding a length estimate of 7–8 m for this individual (Madsen, 1976).

Chevron DMNH 2013-07-1990 is assigned to Theropoda due to the presence of paired anterior processes on the base, while a tetanuran affinity is supported by the posterior curvature of the shaft (Rauhut, 2003). Paired anterior and posterior processes are observed across a range of tetanurans including *Torvosaurus*, *Allosaurus*, *Acrocanthosaurus*, *Neovenator*, *Tyrannosaurus*, *Alioramus*, and *Daspletosaurus* (Brochu, 2003; Brusatte, Benson & Hutt, 2008; Currie & Carpenter, 2000; Harris, 1998). It differs from tyrannosauroids, which possess an enlarged haemal canal, widely spaced and diminutive anterior processes, and straight shaft with a posteriorly expanded and spatulate distal end (Brochu, 2003). This specimen is most similar to the mid-caudal chevrons of allosauroids, particularly *Allosaurus*, which are curved and distally unexpanded with prominent, closely spaced anterior processes (Madsen, 1976). In particular posteriorly curved, transversely compressed chevrons with unexpanded or slightly expanded distal ends are characteristic of carcharodontosaurs including *Acrocanthosaurus*, *Concavenator*, *Neovenator*, and *Siats* (Brusatte, Benson & Hutt, 2008; Cuesta, Ortega & Sanz, 2019; Currie & Carpenter, 2000; Zanno & Makovicky, 2013). However, DMNH 2013-07-1990 differs from many tetanurans in possessing a more flattened and rectangular articular surface, a feature also present in *Siats meekerorum* (pers. obs.; Fig. 3). DMNH 2013-07-1990 is close in size to the fifteenth chevron of *Allosaurus* (length about 230 mm), which belongs to an individual approximately 7–8 m in length (Madsen, 1976).

COELUROSAURIA von Huene, 1914

TYRANNOSAUROIDEA Osborn, 1905

Referred material–DMNH 2013-07-1701 and SMU 77218

***Description:***

**Figure 4:**
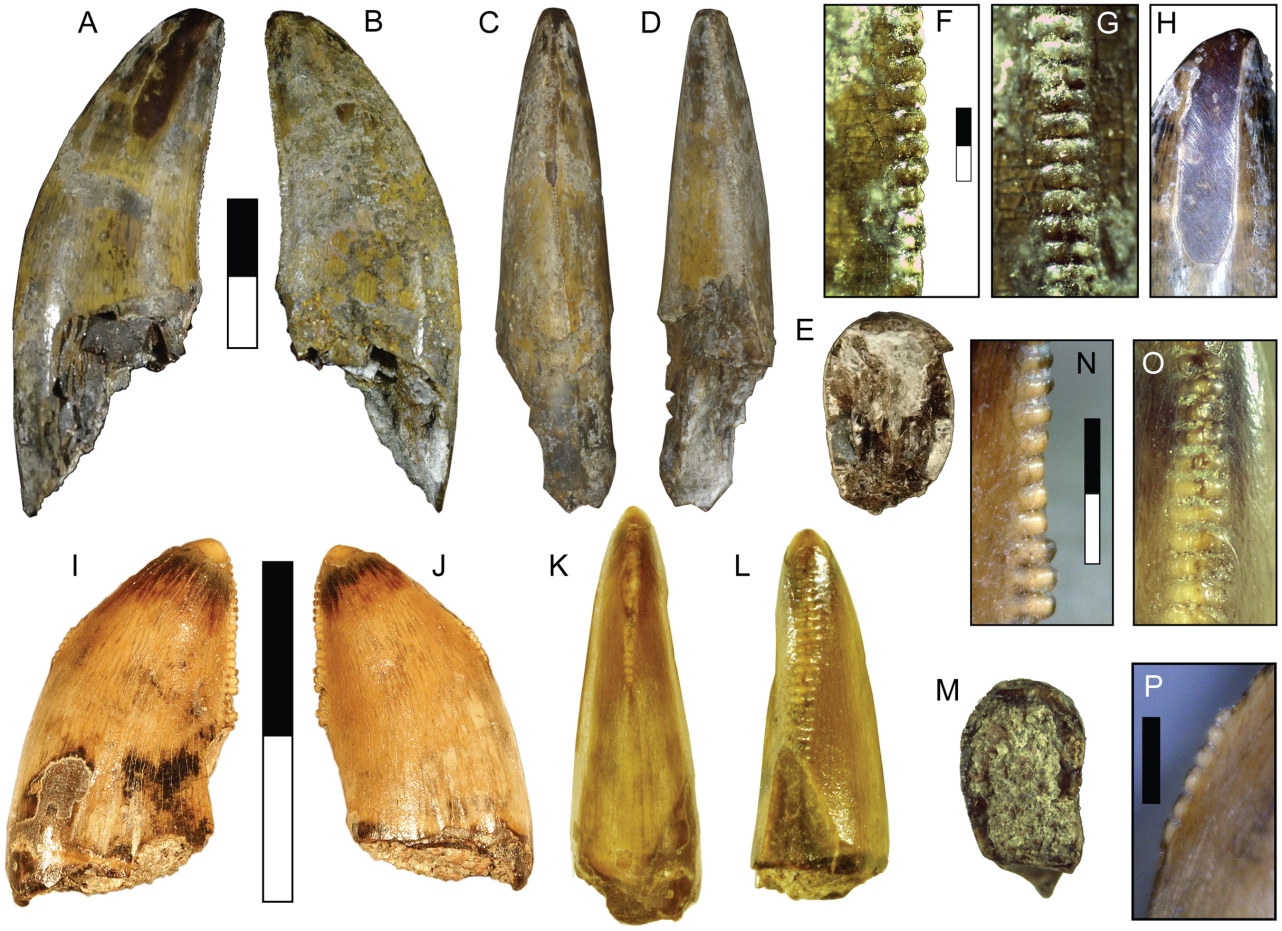
Teeth assigned to Tyrannosauroidea. DMNH 2013-07-1701 shown in lingual (A), labial (B), mesial (C), distal (D), and basal (E) views, distal denticles in lateral (F) and distal (G) views, and detailed view of apical wear facet (H). SMU 77218 shown in labial (I), lingual (J), mesial (K), distal (L), and basal (M) views, distal denticles in lateral (N) and distal (O) views, mesial denticles in lateral view (P). Scale bars of unbordered images are 10 mm. Scale bars of bordered images are 1 mm, except P which is 0.5 mm.

DMNH 2013-07-1701 is a well-preserved tooth with a damaged base and partial root (Fig. 4; Table 1). SMU 77218 is a smaller crown with enamel spalling on the apex and missing a small portion of the base. This morphotype is stout and moderately recurved with a wide oval base (average CBR = 0.58 after reconstruction: Table 2). The mesial carina is moderately convex and possesses a slight lingual curvature approaching the base. The distal carina is mildly concave and extends to the base, with a distinct labial curve midway along its length. Mesial denticles are incompletely preserved and those observed are small (0.22 mm MDL: Table 3) and rounded, extending only about half the height of the crown. Distal denticles are large (0.32–0.33 mm DDL: Table 3) and subquadrangular to subrectangular in shape with a symmetrical, convex margin. The interdenticle space is wide and deep, extending into a wide interdenticular slit between adjacent denticles. The basal surface of SMU 77218 possesses three low, longitudinal ridges on both labial and lingual sides that converge apically. DMNH 2013-07-1701 preserves a wear facet on the lingual apex that is 9.3 mm tall apicobasally and 2.2 mm wide mesiodistally. The wear facet is rounded basally, overlaps the apical end, and its surface contains parallel striations offset at a 45° angle from the long axis of the facet. The enamel surface texture is irregular or smooth.

***Comparisons:***

These teeth share numerous apomorphies with tyrannosauroids, including robust incrassate crowns (CBR > 0.58: Table 2), rounded or oval base, and large, chisel-shaped, widely spaced denticles (Brusatte et al., 2010; Williamson & Brusatte, 2014). A basal lingually deflected mesial carina is observed in the teeth of some tyrannosauroids including *Appalachiosaurus* (Carr, Williamson & Schwimmer, 2005), the juvenile *Tyrannosaurus* BMRP 2002.4.1 (“Jane”; pers. obs.), and isolated tyrannosaurid teeth from the Judith River Group (Sankey et al., 2002). A labial curve on the distal carina is observed on some teeth of BMRP 2002.4.1 (pers. obs.), *Dryptosaurus* (Brusatte, Benson & Norell, 2011), and isolated tyrannosauroid teeth from Cenomanian-Turonian deposits of Uzbekistan, some of which are referred to the non-tyannosaurid tyrannosauroid *Timurlengia* (Averianov & Sues, 2012; Brusatte et al., 2016). Wear facets on occlusal surfaces are a unique feature of tyrannosaur teeth, where they are found isolated to labial/lingual sides, are elliptical in shape, are uniformly flat, and contain sets of parallel striations offset 15° from the long axis of the facet (Schubert & Ungar, 2005). The wear facets fit these criteria. The incrassate shape and robust denticle morphology places these specimens as a more derived member of the clade, however the greater angle of the striations suggests this taxon falls outside Tyrannosauridae (Brusatte et al., 2010). As incrassate morphology exists predominantly in mature teeth, crown height suggests this taxon may have been only a medium-sized predator, with an estimated length of 2.7–4.8 m (Table 3).

COELUROSAURIA von Huene, 1914

ORNITHOMIMOSAURIA Barsbold, 1976

Referred material–SMU 76809

***Description:***

**Figure 5:**
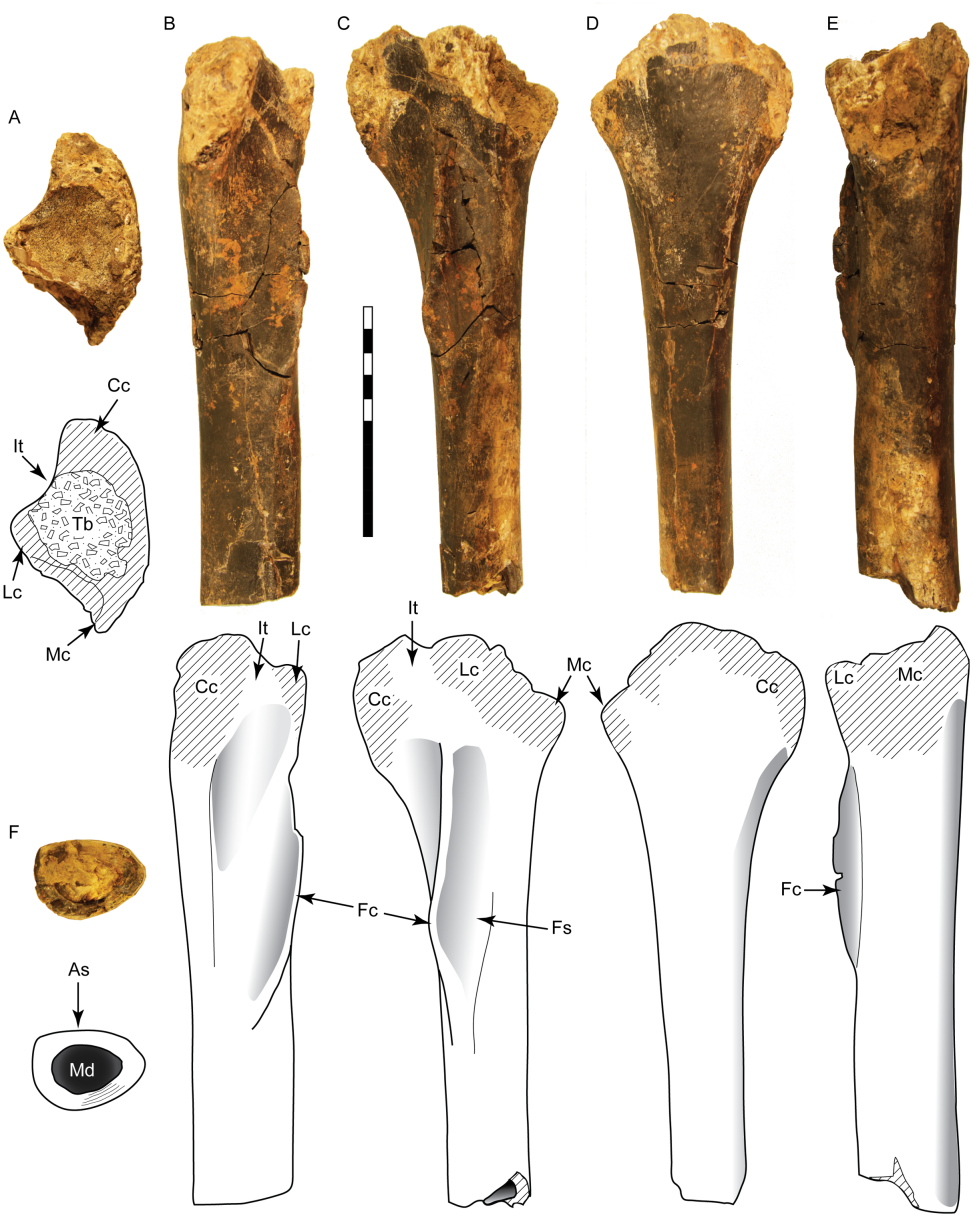
Tibia SMU 76809 assigned to Ornithomimosauria. Shown in proximal (A), anterior (B), lateral (C), medial (D), posterior (E), and distal (F) views. As, anterior surface; Cc, cnemial crest; Fc, fibular crest; Fs, articular surface for fibula; It, incisura tibialis; Lc, lateral condyle; Mc, medial condyle; Md, medullary cavity; Tb, exposed trabecular bone. Scale bar is 10 cm.

The specimen consists of the proximal half of a left tibia, missing both condyles and a portion of the cnemial crest, with a preserved length of 265 mm (Fig. 5). In proximal view the medial surface of what would form the medial condyle is gently arched, curving laterally to form the medial border of the cnemial crest. The posterior margin of the shaft slopes smoothly into the surface of the medial condyle. The preserved portion of the cnemial crest is robust and starts off parallel to the shaft, gently sloping anteriorly and curving laterally as it approaches the condyles. The lateral surface of the cnemial crest is concave, while the medial side is convex. The fibular crest is low and elongate (85 mm long) and clearly separate from the condyles. In lateral view it is straight proximally and then begins to curve anteriorly at its midpoint, where it peaks. The shaft is straight and the broken distal end occurs approximately midshaft, where it has an anteroposterior length of 36 mm and mediolateral width of 49 mm. At this level the shaft is flattened anteriorly, convex posteriorly, and anteroposteriorly deeper medially than laterally, creating a D-shaped cross-section. With an estimated complete tibial length between 560 and 600 mm, the ratio of length to midshaft (mediolateral) width would be 12:1 and the tibial length to midshaft (anteroposterior) length would be about 17:1 (Table S1).

***Comparisons:***

SMU 76809 can be placed in the Tetanurae due to possessing a fibular crest separate from the condyles (Rauhut, 2003). It lacks characters seen in the Tyrannosauroidea and Allosauroidea, including distinct concavities distal to the condyles and enlarged, distally placed fibular crest (Brusatte, Benson & Norell, 2011; Brusatte, Benson & Hutt, 2008; Carr, Williamson & Schwimmer, 2005; Madsen, 1976; White et al., 2013). This specimen contains a number of characters found in ornithomimosaurs, including a flat posterior margin distal to the condyles in lateral view, a laterally curved cnemial crest, D-shaped midshaft cross-section, and a rounded, proximally-placed fibular crest (Allain et al., 2014; Brownstein, 2017a; Buffetaut, Suteethorn & Tong, 2009). An elongated and low fibular crest is noted as a feature unique to ornithomimids (McFeeters et al., 2016; Sues & Averianov, 2016). An anterior curvature of the distal fibular crest is visible in a partial ornithomimid tibia from the Campanian Blufftown Formation of Georgia (Schwimmer et al., 1993). Midshaft ratios indicate SMU 76809 is gracile, particularly in the midshaft length, with proportions similar to other derived ornithomimids (Fig. S1). However, without the confirmation of additional ornithomimid characters from the incomplete proximal and distal ends, this specimen is here assigned to the Ornithomimosauria. Compared to the tibiae of ornithomimids, SMU 76809 is similar to *Gallimimus* and *Struthiomimus* (tibial lengths 737 and 534 mm, respectively) with an estimated mass of 175–450 kg (Christiansen & Fariña, 2004).

COELUROSAURIA von Huene, 1914

MANIRAPTORA Gauthier, 1986

DROMAEOSAURIDAE Matthew & Brown, 1922

Referred material–DMNH 2013-07-1080, DMNH 2014-06-02, SMU 73779, SMU 76948, SMU 77217

***Description:***

**DMNH 2013-07-1080 and DMNH 2014-06-02**

**Figure 6:**
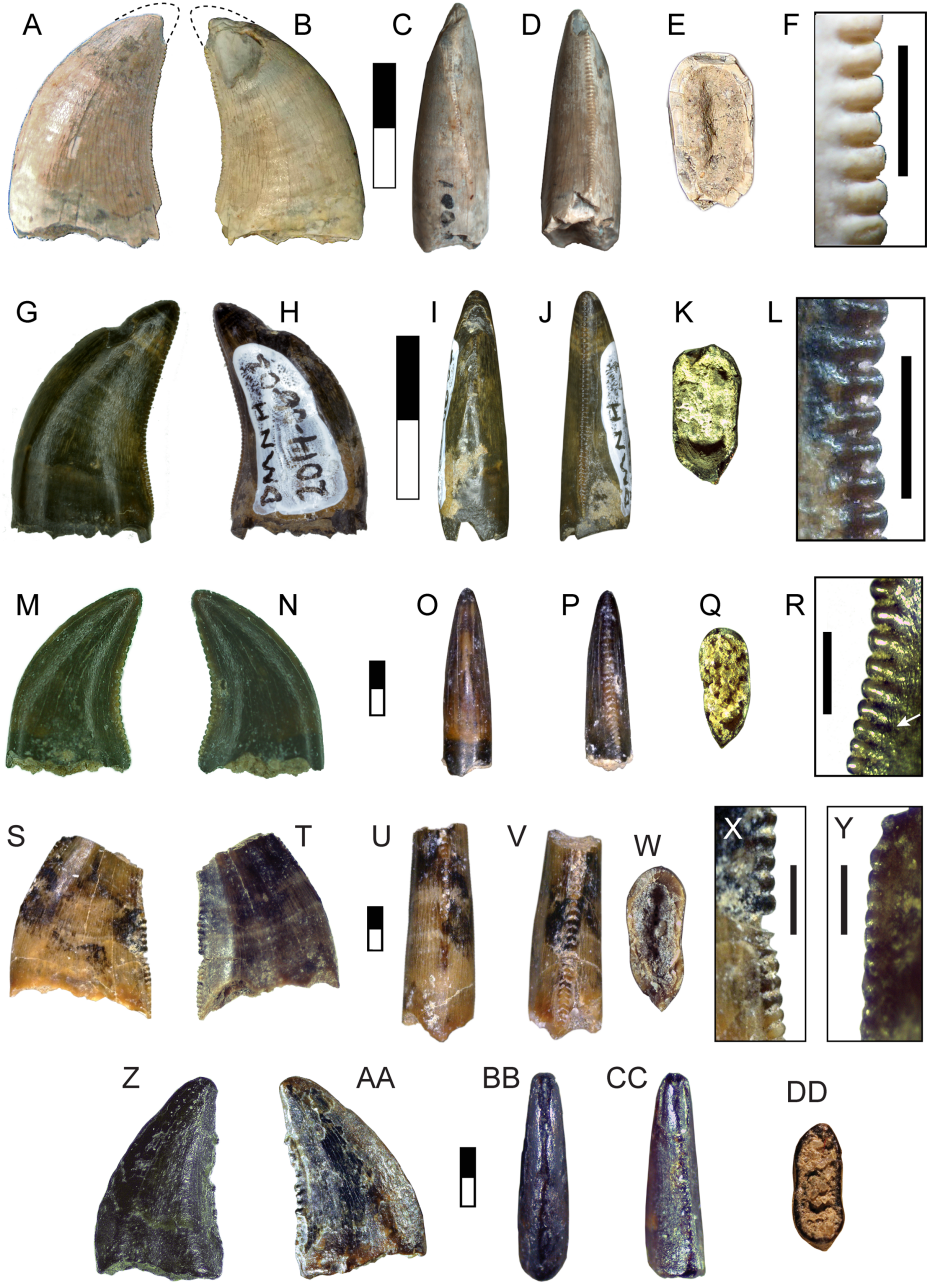
Teeth assigned to Dromaeosauridae. DMNH 2013-07-1080 shown in lingual (A), labial (B), mesial (C), distal (D) and basal views (E); lateral view of distal denticles (F). DMNH 2014-06-02 shown in lingual (G), labial, (H), mesial (I), distal (J), and basal (K) views; lateral view of distal denticles (L). SMU 73779 shown in lingual (M), labial (N), mesial (O), distal (P) and basal views (Q); lateral view of distal denticles (R). SMU 77217 shown in labial (S), lingual (T), mesial (U), distal (V), and basal (W) views; lateral view of mesial denticles (X) and distal denticles (Y). SMU 76948 shown in lingual (Z), labial (AA), mesial (BB), distal (CC), and basal (DD) views. White arrow indicates basally-oriented interdenticular sulci. For (A–L), scale bars of unbordered images are 10 mm and bordered images are 1 mm. For (M–DD), scale bars of unbordered images are 1 mm and bordered images are 0.5 mm.

These teeth are broad and triangular in lateral view, and distally recurved (Figs. 6A, 6B; Table 2). The base lacks any constriction and is moderately laterally compressed with a subrectangular cross-section. Distinct mesial and distal carinae are present, each possessing denticles. The mesial carina is convex, aligned to the midline apically and bears a distinct lingual deflection that terminates just above the cervix. The distal carina extends basally beyond the cervix, is straight and positioned labially. Distal denticles are small (3.5 per mm) subrectangular with a symmetrical convex margin and a mild apical inclination. The interdenticular space is wide and shallow while the interdenticular diaphysis is closed. Mesial denticles are very reduced (approximately 4 per mm), appearing only as a series of raised, lenticular bumps that extend half the length of the carina. A wide, apicobasally elongate depression is present on the base of the lingual surface. DMNH 2013-07-1080 exhibits enamel spalling on the apex. The enamel texture is braided.

**SMU 73779, SMU 76948, and SMU 77217**

While strongly similar to the above, these specimens differ in a few key respects (Figs. 6C–6E). These teeth are much smaller, with a CH between 3.2–5 mm. The base has a more oval or rounded shape and figure-eight outline. These teeth contain distinct anterior, central, and posterior facets on labial and lingual sides. Both carinae are well developed and strongly curved. The mesial carina extends nearly to the base and possesses a mild lingual deviation. Mesial denticles are present only in SMU 77217, being small and rounded in shape. Distal denticles are larger, starting as subrectangular basally and transitioning to a lower subquadrangular shape near the apex. These denticles have a symmetrical, convex margin. Basally oriented interdenticular sulci are present between the lowest 10 denticles of SMU 73779. The enamel texture is braided.

***Comparisons:***

These teeth possess a number of dromaeosaurid characters, including a strongly concave distal margin, lack of basal constriction between root and crown, smaller mesial than distal denticles, and a lingual concavity along the basal surface (Evans, Larson & Currie, 2013; Hendrickx, Mateus & Araújo, 2014; Hendrickx et al., 2019; Turner, Makovicky & Norell, 2012). An identification of Velociraptorinae can be excluded because distal denticles are subequal in size to mesial denticles (low DSDI: Table 2), falling below the range identifying velociraptorines (Brinkman, Cifelli & Czaplewski, 1998; Currie, 1995; Currie, Rigby & Sloan, 1990; Currie & Varricchio, 2004; Gascó et al., 2012; Larson, 2008). DMNH 2013-07-1080 and DMNH 2014-06-02 are distinct in possessing a lingually deflected mesial carina, which is an apomorphy of dromaeosaurine teeth (Currie, Rigby & Sloan, 1990; Hendrickx et al., 2019; Kirkland, Gaston & Burge, 1993; Larson, 2008; Rauhut, 2002). While the teeth of some tyrannosauroids demonstrate a similar deflection, it is located basally (Carr, Williamson & Schwimmer, 2005; Sankey et al., 2002; Xu et al., 2006), whereas here the deflection occurs midway up the mesial carina. DMNH 2013-07-1080 is larger than most dromaeosaurid teeth, exceeded in CH only by the teeth of *Utahraptor* (Kirkland, Gaston & Burge, 1993), *Dakotaraptor* (DePalma et al., 2015), and *Achillobator* (Perle, Norell & Clark, 1999), with an estimated length of over 5 m (Table 3).

Originally described by Lee (1997a), SMU 73779 was attributed to *Richardoestesia* based on its superficial similarity to specimens described from the Judith River Formation (Currie, Rigby & Sloan, 1990). As subsequent work has noted, it is difficult to differentiate the teeth of small dromaeosaurids from *Richardoestesia* morphotypes (Longrich, 2008). These teeth differ from *Richardoestia* in having a more concave distal margin, chisel-shaped distal denticles, and interdenticular sulci between basal denticles. While some teeth are similar in denticle density to *Richardoestesia*, the similarity is likely due to small size (D’Amore & Blumenschine, 2012). Basal denticles with interdenticular sulci are also observed in *Dromaeosaurus* (Currie, Rigby & Sloan, 1990), and all teeth possess the synapomorphies of Dromaeosauridae noted above. These specimens are therefore assigned to the Dromaeosauridae. Denticle size yields an estimated body length of 1.4–1.5 m (Table 3).

COELUROSAURIA von Huene, 1914

MANIRAPTORA Gauthier, 1986

TROODONTIDAE Gilmore, 1924

Referred material–SMU 76947

***Description:***

**Figure 7:**
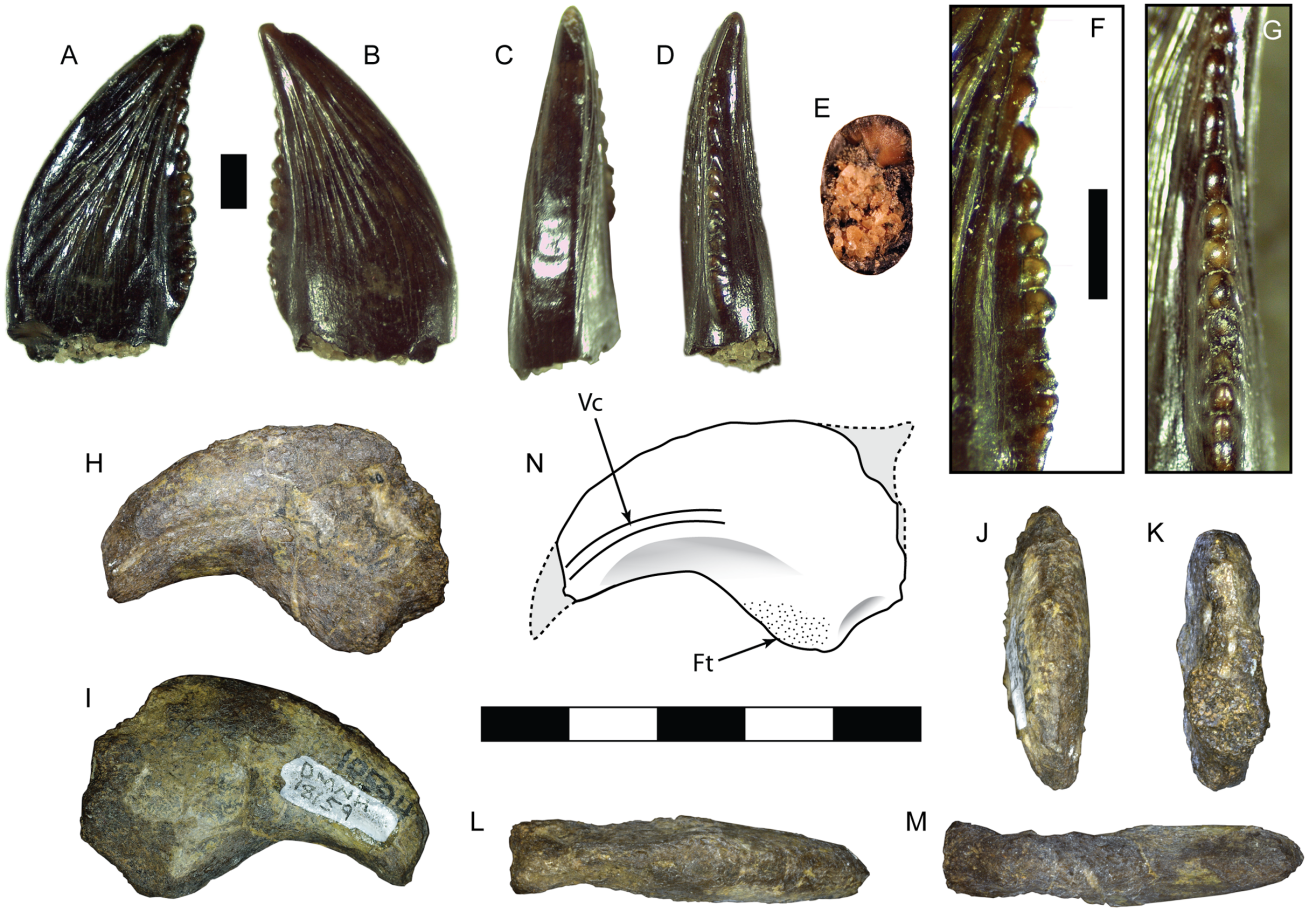
Other maniraptoran material. Tooth SMU 76947 assigned to Troodontidae shown in labial (A), lingual (B), mesial (C), distal (D), and basal (E) views; distal denticles shown in lateral (F) and distal (G) views. Manual ungual DMNH 18159 shown in lateral (H–I), distal (J), proximal (K), dorsal (L), and ventral (M) views; reconstruction of complete ungual (N). Ft, flexor tubercle; Vc, neurovascular canal. Scale bar in A–G is 0.5 mm and scale bar in H–N is 5 cm.

This tooth morphotype is represented by a single well-preserved, complete crown with a distinctly folidont shape (Fig. 7A). The base is bulbous with a constriction and semicircular cross-section. The lingual surface is flattened while the labial surface is strongly convex. Elongate fluted ridges extend from base to apex on both sides, with some anastomosing as they approach the apex. Both carinae are placed towards the lingual surface of the crown. The mesial carina lacks denticles, is convex and projects mesiolingually as a prominent ridge. The distal carina is mildly convex with an asymmetric profile and offset on a separate shelf for a majority of its length. Distal denticles are enlarged, with only 11 present on the crown. Denticle shape is heterogenous, starting as subrectangular with a parabolic margin at the base and increasing in size apically. The four most apical denticles are larger, apically oriented and bulbous in shape, with a wide interdenticular space that is continuous with a short, basally oriented sulcus. The denticles are rounded in distal view, lacking the sharp edge typically seen on many theropod teeth. The enamel texture is irregular or smooth.

***Comparisons:***

This specimen shares numerous characters in common with members of the Troodontidae including a bulbous base, basal constriction, nearly circular basal cross-section, and large apically inclined denticles that increase in size apically (Currie, Rigby & Sloan, 1990; Sankey et al., 2002; Turner, Makovicky & Norell, 2012). One trait in particular, the low denticle count, is recognized as a synapomorphy of the Troodontidae (Turner, Makovicky & Norell, 2012). However, this specimen contains an unusual combination of features observed separately among different troodontid taxa. Distal denticles are rounded and lacking the distinctive apical hook. A majority attach to the carina perpendicularly, a feature seen in teeth of *Pectinodon* (Longrich, 2008). Longitudinal ridges occur in *Paronychodon* and *Zapsalis*, two morphotypes usually associated with the Troodontidae and in SMU 76947 the longitudinal ridges anastomose as in *Paronychodon* (Larson, 2008; Sankey et al., 2002), however this specimen possesses denticles. A mesiolingually directed mesial carina is observed in isolated troodontid teeth from the Cenomanian of Uzbeckistan, Santonian of Tajikistan (Averianov & Sues, 2007), and Campanian- Maastrichtian of New Mexico (Williamson & Brusatte, 2014). This feature is also seen in premaxillary and anterior dentary teeth of *Pectinodon* (Longrich, 2008). The unique combination of features suggests that this morphotype represents a new taxon, adding to the morphological diversity of troodontid teeth. Body length estimates for troodontids are complicated by their unusual denticle morphology, leading to an overestimate (D’Amore & Blumenschine, 2012). Therefore, this specimen was compared to *Geminiraptor*, which possesses alveoli with the same dimensions, yielding a revised body length estimate of about 2 m (Senter et al., 2010).

COELUROSAURIA von Huene, 1914

MANIRAPTORA Gauthier, 1986

Referred material–DMNH 18159

***Description:***

This partial ungual is missing most of the distal tip and articular facet, including a small portion of the proximodorsal surface (Fig. 7B). Its external surface is strongly weathered, but patches of original bone surface remain. Despite being partially crushed, it shows a high degree of mediolateral compression. The preserved portion is 40 mm long, 26 mm tall, and 8 mm wide with a broad, weakly incised vascular groove that terminates at broken edges on the proximal and distal ends. In lateral view the dorsal surface is arched, indicating that the ungual was strongly recurved. The flexor tubercle is large and bulbous, with a height of 9.3 mm. Distally the flexor tubercle slopes up gently to meet the ventral surface of the ungual body, while the proximal end demonstrates only a slight convexity, forming a shallow sulcus. The preserved distal portion is moderately curved and narrow. Both dorsal and ventral surfaces of the distal end are 6 mm wide, where the dorsal surface is strongly arched and the ventral surface is flat or weakly convex. The mediolateral compression, enlarged flexor tubercle, and high degree of curvature are all consistent with a manual element.

***Comparisons:***

DMNH 18159 shares similarities with a variety of coelurosaurs. Even though the articular facet is not preserved, the height of the flexor tubercle is over half the remaining ungual height. Because the articular facet in theropod manual unguals rarely extends fully between the dorsal and ventral surfaces of the ungual body, the actual size of the articular facet was likely less, further increasing the ratio. An enlarged flexor tubercle is a character common across maniraptorans, particularly paravians (Rauhut, 2003). The flexor tubercle itself is relatively low and rounded and is distally inset, unlike the unguals of deinonychosaurs, which possess an enlarged, pendulous flexor tubercle that is posteriorly retracted and separated from the ventral edge of the articular facet by a transverse groove (Turner, Makovicky & Norell, 2012). Among maniraptorans this ungual is most similar to phalanx I-2 or II-3 of caenagnathids, whose unguals are mediolaterally compressed, strongly curved, and possess a large, distally positioned flexor tubercle (Bell, Currie & Russell, 2015; Funston et al., 2015). However, the specimen cannot be confidently assigned to the Caenagnathidae without the confirmed presence of a proximodorsal lip.

COELUROSAURIA von Huene, 1914

Referred material–SMU 73778, SMU 77213, SMU 77214

***Description:***

**Figure 8:**
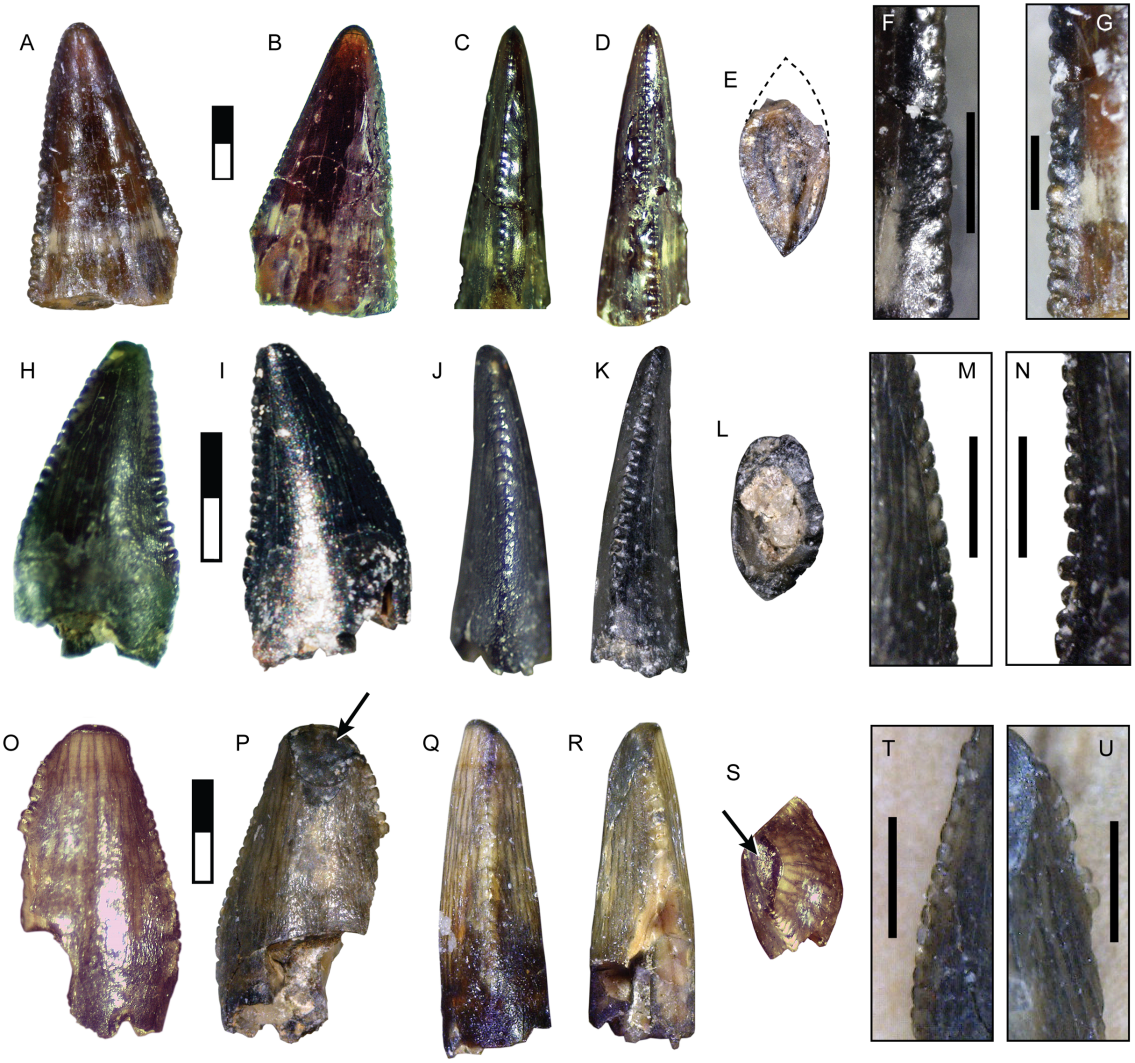
Teeth assigned to Coelurosauria indet. SMU 77213 shown in labial (A), lingual (B), mesial (C), distal (D), and basal (E) views; lateral view of mesial denticles (F) and distal denticles (G). SMU 73778 shown in labial (H), lingual (I), mesial (J), distal (K), and basal (L) views; lateral view of mesial denticles (M) and distal denticles (N). SMU 77214 shown in labial (O), lingual (P), mesial (Q), distal (R), and apical (S) views; lateral view of mesial denticles (T) and distal denticles (U). Black arrows indicate spalled enamel surface. Scale bars of unbordered images are 1 mm. Scale bars of bordered images are 0.5 mm.

Teeth of this morphotype are small, with CH between 2–5 mm (Table 2). Lingual and labial surfaces are difficult to distinguish due to the unusual morphology (Figs. 8A–8C). These teeth have a nearly triangular, recurved folidont shape with a lenticular or parlinon cross-section and evidence of a narrower root. The apex is rounded with few or no denticles and is mesially concave in some specimens. The apex of SMU 77214 shows enamel spalling. Both carinae are relatively straight and extend nearly to the base and are oriented more towards one side of the tooth than the other. Being nearly identical, carinae are identified here based on difference in denticle morphology: the side possessing the larger denticles is considered to be the distal edge. The mesial denticles are low and rounded with MDL of 0.085–0.106 mm (Table 3). Distal denticles are subquadrangular with a slight apical incline and DDL of 0.10–0.15 mm (Table 3). Mesial denticles tend to be apicobasally longer than the distal ones leading to DSDI values less than 1 (Table 2). The basal quarter or third of the tooth lacks enamel but excludes the carinae. The enamel texture is smooth or irregular (*sensu* Hendrickx et al., 2019)

***Comparisons:***

SMU 73778 was previously described by Lee (1997a) and assigned to cf. *Richardoestesia*. These teeth share several characters identified in specimens assigned to the form taxon *Richardoestesia*, including possessing small rounded denticles, denticles subequal in size, well-developed facets, moderately recurved or upright shape, lenticular cross-section, inward curvature of the crown, extension of both carinae towards the base of crown, slight sigmoidal curvature or mesially concave apex, a root narrower than crown, and a large area lacking enamel at the base of the tooth (Averianov & Sues, 2019; Larson & Currie, 2013; Longrich, 2008; Williamson & Brusatte, 2014). In particular, these teeth show a strong similarity to *R. asiatica*, but are smaller (Averianov & Sues, 2019). A smooth or irregular enamel texture is a synapomorphy of Neocoelurosauria (Hendrickx et al., 2019). Given the many uncertainties regarding the affinities and usage of the taxon *Richardoestesia* (Averianov & Sues, 2019; Longrich, 2008), these specimens are assigned to an indeterminate small coelurosaur approximately 1.2–1.6 m in length (Table 3).

THEROPODA Marsh, 1881

Referred material–DMNH 2013-07-1082, DMNH 2013-07-0492

***Description:***

**DMNH 2013-07-1082**

**Figure 9:**
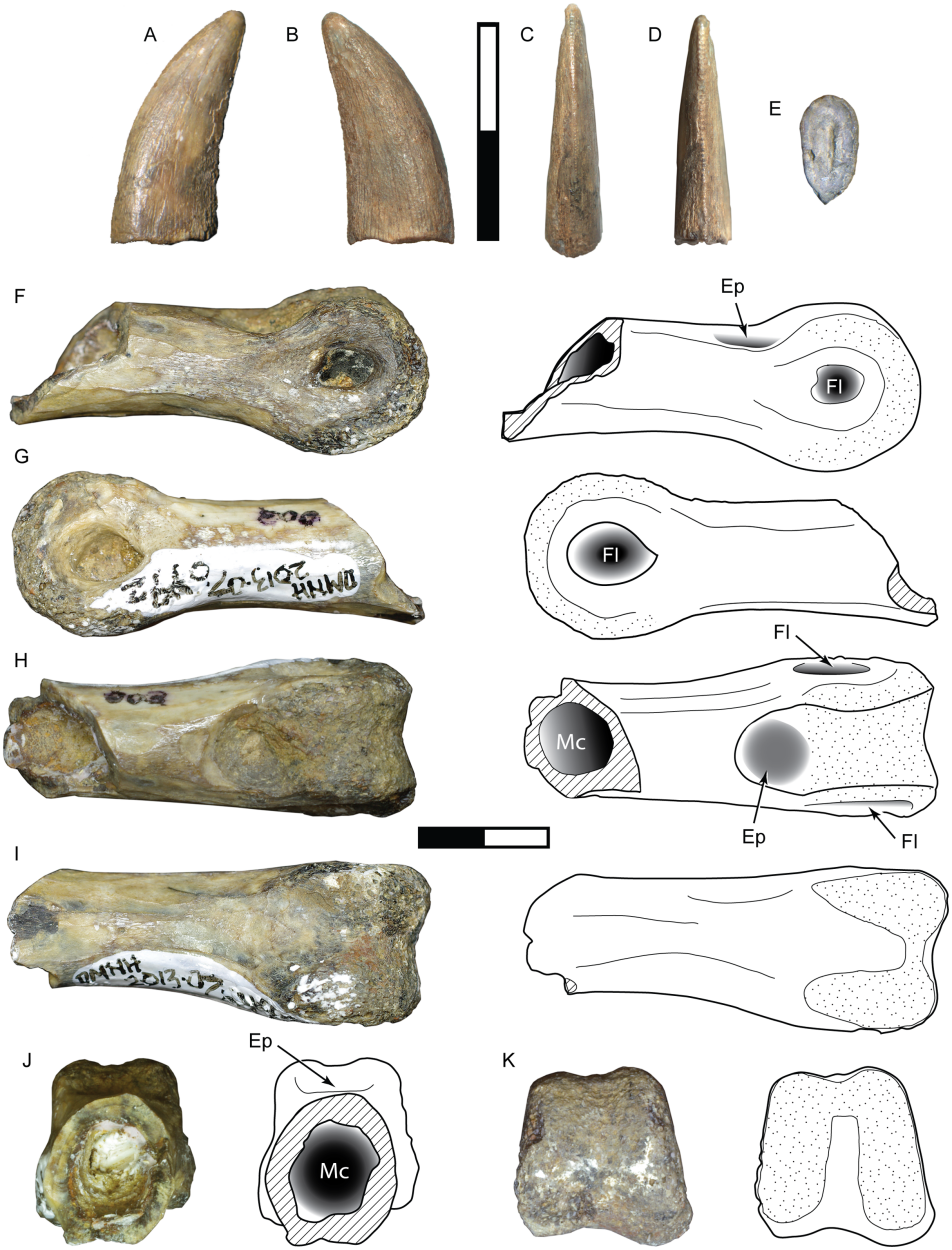
Specimens assigned to Theropoda indet. Tooth DMNH 2013-07-1082 shown in labial (A), lingual (B), mesial (C), distal (D), and basal (E) view. Pedal phalanx DMNH 2013-07-0492 shown in lateral (F–G), dorsal (H), ventral (I), proximal (J), and distal (K) views. Ep, extensor pit; Fl, ligament fossa; Mc, medullary cavity. Scale bars are 10 mm.

This morphotype is represented by a single tooth (Fig. 9A; Table 1). The enamel surface is weathered and a majority of the denticles damaged or missing making assignment to any particular group problematic. The tooth is small (CH = 10.60 mm: Table 2), mildly recurved, and labiolingually compressed with an oval base. There is some enamel spalling at the apex. The mesial carina is gently convex and ends before reaching the base, moving towards the lingual side while maintaining a straight course. Only the bases of mesial denticles are visible, showing they were small with a MDL of 0.21 mm (Table 3). The distal carina is mildly concave and located centrally along the posterior surface. Distal denticles are larger than mesial denticles (DDL of 0.22 mm: Table 3). The weathered state of the tooth makes their complete form difficult to determine, but the distal denticles are most likely chisel-shaped and show they are apically inclined. Denticle size difference is among the highest in the sample (DSDI = 1.44: Table 2).

**DMNH 2013-07-0492**

This partial phalanx is missing the proximal epiphysis and an unknown amount of the diaphysis but exposes a hollow medullary cavity with an extremely thin cortical layer (Fig. 9B). It is uncertain whether this is a manual or pedal element. The total preserved length is 32 mm. The shaft has a mediolateral width of 8.8 mm and dorsoventral height of 9.2 mm. The shaft shows signs of slight widening proximally, and thus may belong to a shorter phalanx. The distal articular surface is strongly ginglymoid and symmetrically rounded in lateral view, measuring 11.8 mm wide and 11 mm tall. A shallow, semicircular extensor fossa extends proximally from the distal articular surface. A deeply excavated and teardrop-shaped collateral ligament pit is centered on each side of the distinct condyles. Both are features of a middle or distal nonungual phalanx.

***Comparisons:***

The tooth exhibits features in common with both the carcharodontosaur and tyrannosauroid morphotypes described above, but its small size and lack of certain key elements such as denticle shape and enamel surface features precludes confident assignment to either taxon. It may represent a juvenile form of one, with its ontogenetic state masking its identity, or belongs to a distinct taxon. Body length is estimated at about 2.5–2.6 m (Table 3).

The narrow, elongate dimensions and thin cortex of the partial phalanx suggest a more gracile animal, and bears the most similarity to the phalanges of ornithomimosaurs and paravians (Turner, Makovicky & Norell, 2012).

***Morphometric analysis:***

**Figure 10:**
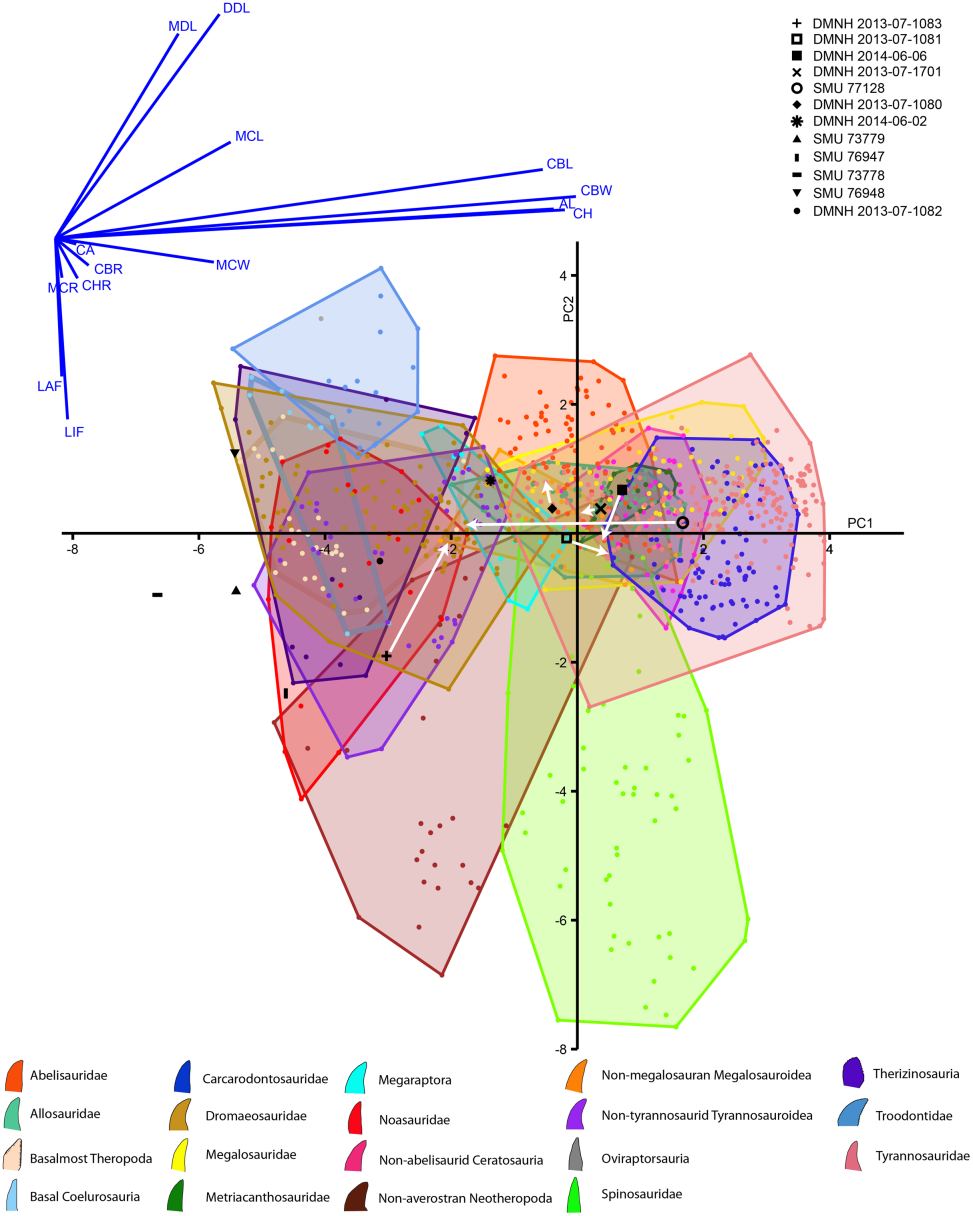
Principal components plot and loadings biplot for known and unknown teeth combined. Graph axes represent the two principal components (PCs) with the greatest amount of variance. Known teeth from published data sets are colored and represented by the legend at the bottom, and unknown teeth reported here are in black with the legend at the top. Arrows indicate where the teeth with missing data move when fully reconstructed (Note the PC scores for most teeth were different for the observed and reconstructed analyses, but, because these changes were so small, they could not be seen between the two PC plots. We therefore combined them into one, and only show movement of reconstructed teeth).

The discriminant analysis produced principal components, the first two of which represent over 72% of the overall variance of both the Lewisville Formation and published teeth combined (Fig. 10, Supplemental Materials). PC1 is heavily influenced by the overall size of the crowns, with CBL, CBW, CH, and AL strongly influencing it positively. Mid-crown measures also positively weighed into the axis, but to a lesser extent. PC2 was composed of positive loadings of denticle heights, and negative loadings of both labial and lingual flutes. Large taxa like tyrannosaurids, carcharodontosaurids, and non-abelisaurid ceratosaurs exist entirely in the positive region of PC1, whereas smaller taxa such as dromaeosaurs, basal coelurosaurs, noasaurs, therizinosaurs, basal-most theropods, and non-tyrannosaurid tyrannosauroids are exclusive to the negative region. Most taxa possess teeth on both the positive and negative sides of PC2, with the exception of troodontids being entirely positive and spinosaurids and non-averostran neotheropods being primarily negative.

Concerning the Lewisville Formation crowns reported here, the smallest five teeth (SMU 73778, 73779, 76947, 76948, and DMNH 2013-07-1082) tended to plot below zero concerning PC1 (Fig. 10, Supplemental Materials), with SMU 76948 having positive PC2 values and all the others having negative. These teeth were categorized as either noasaur or therizinosaur in clade (Table 4). DMNH 2014-06-02 was also fully intact and was categorized as a megaraptoran. The remaining teeth were missing data due to damage, and, when not reconstructed, were assigned as basalmost theropods, an abelisaurid, and a non-megalosauran megalosauroid. When reconstructed these teeth moved noticeably across the morphospace. This caused their clades to change as well for all teeth except DMNH 2013-07-1080, which remained a non-megalosauran megalosauroid. The remainder fall out as metriacanthosaurids, a dromaeosaurid, a therizinosaur, and a non-megalosauran megalosauroid (Table 4).

**Table 4:** Clade Assignments of theropod teeth based on Discriminant Function Analysis.

Tooth	Observed data	Reconstructed data
DMNH2013-07-1083	Basalmost Theropoda	Dromaeosauridae
DMNH2013-07-1081	Basalmost Theropoda	Metriacanthosauridae
DMNH2014-06-06	Abelisauridae	Metriacanthosauridae
DMNH2013-07-1701	Basalmost Theropoda	Non-megalosauran Megalosauroidea
SMU77218	Basalmost Theropoda	Therizinosauria
DMNH2013-07-1080	Non-megalosauran Megalosauroidea	Non-megalosauran Megalosauroidea
DMNH2014-06-02	Megaraptora	Megaraptora
SMU73779	Therizinosauria	Therizinosauria
SMU76947	Noasauridae	Noasauridae
SMU73778	Therizinosauria	Therizinosauria
SMU76948	Therizinosauria	Therizinosauria
DMNH2013-07-1082	Noasauridae	Noasauridae

**Note:**

Theropod teeth were placed in one of the 19 clades based on either observed data (left) or reconstructed data with missing measurements estimated (right).

## Discussion

### Theropod diversity and relationships

With the exception of two teeth described by Lee (1997a), non-avian theropod remains from the Lewisville Formation are largely unknown. Though a wide variety of vertebrates are recognized, identifiable fossils are relatively rare and based on predominantly fragmentary and isolated remains (Adams et al., 2011; Adrian et al., 2019; Adrian et al., 2021; Drumheller et al., 2021; Head, 1998; Jacobs & Winkler, 1998; Krause & Baird, 1979; Lee, 1997a; Main, Noto & Weishampel, 2014; Main et al., 2011; McNulty & Slaughter, 1962; McNulty & Slaughter, 1968; Noto et al., 2020; Tykoski & Fiorillo, 2010). The material described here is no exception, yet is sufficient to demonstrate both taxonomic and body size diversity among non-avian theropods in the Lewisville Formation. There are six or seven identifiable taxa present, representing a large carcharodontosaur, a medium-sized tyrannosauroid, an ornithomimosaur, a large dromaeosaurine, a small dromaeosaurid, a small troodontid, and an indeterminate small coelurosaur. These specimens add significantly to the overall taxonomic diversity of the Lewisville Formation.

The fragmentary nature of the material and largely isolated discoveries hinders interpretations of the distribution of theropod taxa within the Lewisville Formation itself. Morphometric analysis of the teeth, while considered de rigueur in theropod tooth descriptions, was largely uninformative, due to the fragmentary nature of the fossils, and returned results inconsistent with the apomorphy-based identifications. This is not wholly unexpected, as the papers upon which we base our methods itself state that these morphometric analyses return significantly overlapping signals, and that they are best used in partnership with phylogenetic or apomorphy-based discussions (Hendrickx, Mateus & Araújo, 2015; Hendrickx, Tschopp & Ezcurra, 2020). We therefore report these results for the sake of completeness, but defer to the apomorphy-based identifications for the remainder of this discussion. Incomplete data weighed heavily on the results of the morphometric analysis (similar to Hendrickx, Tschopp & Ezcurra, 2020). Metrics derived exclusively for our ‘reconstructed’ data changed the clade designation for almost every damaged tooth, and, if our assumptions were indeed accurate, demonstrates the impact of the incomplete data. The published dataset of known teeth is also incomplete not necessarily due to damage, but because teeth came from numerous studies. Certain measurements were excluded for some studies and not others (such as mid-crown measurements and the existence of flutes; see Supplemental Information). Much of the PC output appeared counterintuitive, presumably due to this. For example, SMU 76947 had enlarged distal denticles, no mesial denticles, and folidont shaped crowns that strongly suggest a member of Troodontidae. Although also typical, the existence of flutes was not recorded for most troodontids in the teeth we used from the published dataset, and the inclusion of SMU 76947’s flutes may have placed it along the negative range of PC2 away from the Troodontidae cluster. Many of the teeth classified as noasaursids or therizinosaurs may have been placed there due to their size, even though denticle and enamel characters clearly place them with Dromaeosauridae. Size normalization may reduce this issue, and geometric morphometrics with superimpositions that separate shape from size should be considered in the future (*sensu* D’Amore et al., 2019). Data collection methods also varied in the published data set, with variability in the point along the mesial margin where CBL and AL terminated (for example: Gerke & Wings, 2016 *versus* Hendrickx, Mateus & Araújo, 2015). This may have also influenced the outcome (*sensu* Hendrickx, Tschopp & Ezcurra, 2020).

When considering the recognized clades based on identifiable apomorphies, two potential patterns are noted here. First, large theropods appear more widespread, at least within the study area. Carcharodontosaurian material is found at four separate sites (AAS, Bear Creek, Lake Lewisville, and Veteran’s Park) and the tyrannosauroid teeth occur at two sites (AAS and SMU 245), with both occurring together at two sites (AAS and Bear Creek). Each locality is separated by several kilometers and represents different depositional environments. These large theropods may have ranged widely through the broad delta plain, as many large predators do today (Carbone, Turvey & Bielby, 2011; Pianka & Farlow, 2003). Conversely, smaller theropods appear more restricted, with particular morphotypes confined to individual localities. Dromaeosaurine teeth are found only at the AAS, while the small coelurosaur, dromaeosaurid, and troodontid are currently confined to Bear Creek. This pattern follows the distribution of Lewisville Formation crocodyliforms. Large-bodied taxa like *Deltasuchus* occur at the AAS and Bear Creek and *Terminonaris* is known from AAS, Bear Creek, and Lake Lewisville (Adams, Noto & Drumheller, 2017; Adams et al., 2011; Lee, 1997a; Noto, 2015). The smallest known crocodyliform, *Scolomastax*, is restricted only to the AAS (Noto et al., 2020). However, this difference in distribution between large and small taxa may be due to taphonomic and sampling biases. The widespread presence of larger teeth across depositional environments may be due to their size, differential transport potential, and preservation potential compared to smaller teeth (Peterson, Coenen & Noto, 2014; Wilson, 2008). The extremely small sample size and differences in collection methods (quarrying *vs* surface) at present precludes a quantitative treatment within and between sites.

These discoveries provide new context for which theropod lineages were present in Appalachia at the beginning of the Late Cretaceous. The fossil material described here marks the first record of a large carcharodontosaur allosauroid in Appalachia, consistent with other Cenomanian-aged records for this clade in Laramidia (Krumenacker et al., 2016; Zanno & Makovicky, 2013; Zanno et al., 2019).

The Lewisville Formation records the earliest occurrence of the Tyrannosauroidea in Appalachia. Derived tyrannosauroids may have been present in North America as early as the Albian (Zanno & Makovicky, 2011). Lewisville Formation specimens confirm a relatively early presence on the continent and show the clade was present in western Appalachia during formation of the Western Interior Seaway (Slattery et al., 2015). In eastern Appalachia non-tyrannosaurid tyrannosauroids are represented by a distal metatarsal possibly from the Potomac Formation in New Jersey (Cenomanian), *Appalachiosaurus* from the Demopolis Formation (Campanian) of Alabama and the Coachman Fm. equivalent in South Carolina, *Dryptosaurus* from the Monmouth Group (Campanian-Maastrichtian) of New Jersey, a metatarsus from the Merchantville Formation (Campanian) of Delaware, and isolated tooth crowns from the Marshalltown (Campanian) and Mt. Laurel (Maastrichtian) formations of New Jersey (Baird, 1989; Brownstein, 2017b; Brownstein, 2018a; Brownstein, 2018b; Brownstein, 2019; Carr, Williamson & Schwimmer, 2005; Schwimmer et al., 2015; Weishampel et al., 2004). This study further supports the hypothesis that derived tyrannosauroids in eastern North America may represent a distinct, endemic assemblage and are not the result of western immigrants in the Campanian-Maastrichtian (Carr, Williamson & Schwimmer, 2005).

The presence of dromaeosaurids in the Lewisville Formation is consistent with the Cretaceous record for the group. *Deinonychus* is known from the Early Cretaceous of nearby Oklahoma (Brinkman, Cifelli & Czaplewski, 1998), while dromaeosaurid teeth are known from the Aptian-Albian age Twin Mountains and Antlers Formations of northern Texas and Campanian-Maastrichtian age Aguja Formation of west Texas (Sankey, 2001; Weishampel et al., 2004; Winkler, Murry & Jacobs, 1990). However, those teeth all represent very small individuals, unlike the larger dromaeosaurine teeth described here. Large and small dromaeosaurid teeth are known from Campanian to Maastrichtian deposits of the Atlantic Coastal Plain, including a large dromaeosaurine tooth from North Carolina, large velociraptorine tooth from New Jersey, and small velociraptorine teeth from South Carolina attributed to *Saurornitholestes* (Brownstein, 2018c; Brownstein, 2019; Schwimmer et al., 2015). A small dromaeosaur tooth is also known from the Santonian Mooreville Formation of Alabama (Kiernan & Schwimmer, 2004). This may support the idea that large-bodied dromaeosaurids were a regular component of many terrestrial communities in Appalachia during the Late Cretaceous and possibly coexisted with smaller-bodied dromaeosaurids, however more material will be needed to test this hypothesis (Brownstein, 2019). A second, smaller taxon of dromaeosaur coexisting with a larger form is not uncommon in Cretaceous ecosystems that possess a diverse theropod fauna (Frederickson, Engel & Cifelli, 2018; Gates, Zanno & Makovicky, 2013; Larson, 2008).

Small, unusual coelurosaur teeth (often attributed to *Richardoestesia*) have been noted from a variety of deposits going back as early as the Kimmeridgian, however the only confirmed records of *Richardoestesia* in North America are Santonian to Maastrichtian in age (Antunes & Mateus, 2003; Averianov & Sues, 2019; Currie, Rigby & Sloan, 1990; Larson & Currie, 2013; Longrich, 2008; Sankey, 2001; Williamson & Brusatte, 2014). Isolated coelurosaur teeth attributed to or resembling *Richardoestesia* occur in Early Cretaceous Cloverly, Holly Creek, and lower Cedar Mountain Formations, and Late Cretaceous upper Cedar Mountain, Naturita (“Dakota”) Wayan, and Iron Springs Formations (Cifelli et al., 1999; Eaton et al., 2014; Eaton et al., 1999; Kirkland et al., 1997; Kirkland et al., 1999; Krumenacker et al., 2016; Suarez et al., 2021). The overall similarity between the specimens described here and *Richardoestesia*, suggests the presence of a similar, unusual small theropod in the Lewisville Formation. In Laramidia evidence suggests *Richardoestesia*-like coelurosaurs had a preference for living and feeding in aquatic environments, including a possibly piscivorous diet for these animals (Baszio, 1997; Frederickson, Engel & Cifelli, 2018; Longrich, 2008). Similar small teeth in the Lewisville Formation suggest the same may have been true of coastal plain faunas in Appalachia, though more material is necessary.

Troodontids were previously unknown from Texas and Appalachia, with this specimen representing the easternmost record for the clade in North America. The oldest troodontid in North America, *Geminiraptor*, is known from the Early Cretaceous of Utah (Senter et al., 2010). No teeth are preserved with the specimen, but the alveoli approximately match the size and shape of SMU 76947, suggesting the taxa were similar in size (Senter et al., 2010). A Cenomanian troodontid provides a temporal link between Early Cretaceous taxa and Late Cretaceous forms such as *Pectinodon* and *Paronychodon*. In particular, SMU 76947 shows a strong similarity to *Paronychodon* (morph 6) specimens from the Mussentuchit Member of the Cedar Mountain Formation, further illustrating the faunal similarity between this and the Lewisville Formation (Frederickson, Engel & Cifelli, 2018). The unique mixture of features in SMU 76947 suggests greater taxonomic diversity in Late Cretaceous North American troodontids than is currently known.

The tibia SMU 76809 is consistent with the record of Ornithomimosauria in North America throughout the Cretaceous. Basal ornithomimosaurs were widespread across North America during the Early Cretaceous (Brownstein, 2017a; Galton & Jensen, 1975; Hunt & Quinn, 2018; Ostrom, 1970). Derived ornithomimids become dominant in Appalachia by the Campanian-Maastrichtian (Weishampel et al., 2004). The Lewisville Formation specimen provides a link between the Early and Late Cretaceous records, showing they remained a component of the North American fauna at the beginning of the Late Cretaceous. Unfortunately the fragmentary nature of the specimen does not permit assessment of whether it is a basal member of the clade or is a more derived ornithomimid that began replacing more basal taxa in the Early Cretaceous (Brownstein, 2017a).

### Patterns and comparisons

During the Early Cretaceous North American terrestrial ecosystems were largely cosmopolitan and low-diversity (Zanno & Makovicky, 2011; Suarez et al., 2021). However, during this time large-scale faunal interchange both within Laurasia (EKLInE) and between Laurasia and Gondwana began a significant transition in dominant groups of vertebrates (Amiot et al., 2004; Buffetaut & Ouaja, 2002; Galton & Taquet, 1982; Zanno & Makovicky, 2011). This prolonged exchange influenced the evolution of North American communities, leading ultimately to the assembly of ecosystems that characterize the latter part of the Late Cretaceous (Jacobs & Winkler, 1998; Winkler et al., 1995).

In the Aptian, theropod communities were primarily composed of large allosauroids (carcharodontosaurids), ornithomimosaurs, and dromaeosaurids. In the west the Cloverly and Cedar Mountain (Ruby Ranch Member) Formations contained dromaeosaurids like *Deinonychus*, the large carcharodontosaurid *Acrocanthosaurus*, indeterminate ornithomimosaurs, and the caenagnathid *Microvenator* (D’Emic, Melstrom & Eddy, 2012; Ostrom, 1970; Weishampel et al., 2004). This pattern is largely replicated further east with fossils of *Deinonychus*, *Acrocanthosaurus*, and ornithomosaurs in the Patuxent Formation (Arundel Clay Facies) and Trinity Group of Arkansas (Lipka, 1998).

In the Albian carcharodontosaurs remain part of the community, with notable first appearances of oviraptorosaurs, small dromaeosaurids, and tyrannosauroids in the west, as seen in the Blackleaf (Flood Member), Wayan, and Willow Tank Formations (Bonde et al., 2012; Krumenacker et al., 2016; Ullmann, Varricchio & Knell, 2012). In central-eastern North America the record is similar (with the notable absence of tyrannosauroids) and includes *Deinonychus*, *Acrocanthosaurus*, a *Richardoestesia*-like coelurosaur, small dromaeosaurids, and an ornithomimosaur, as well as large and small theropod tracks in the Holly Creek, Dakota, Twin Mountains, Paluxy, Antlers, and Glen Rose Formations (Suarez et al., 2021; Weishampel et al., 2004; Winkler, Murry & Jacobs, 1989). There are no records of Albian theropods further east.

**Figure 11:**
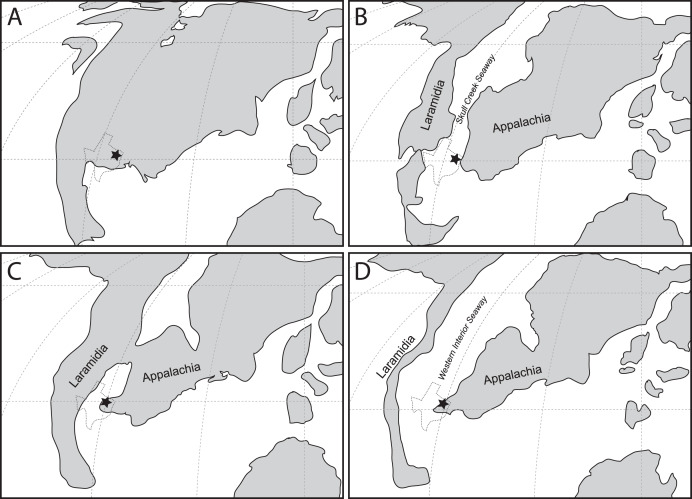
Paleogeographic maps of the mid-Cretaceous interval. (A) Albian, approximately 110 MY. (B) Late Albian, approximately 105 MY, formation of the Skull Creek Seaway (showing reconstruction of Slattery et al., 2015). (C) Early Cenomanian, approximately 100 MY, showing short-term regression established continental connection between Laramidia and Appalachia. (D) middle Cenomanian, approximately 95 MY, showing a continuous Western Interior Seaway. Star marks location of the Lewisville Formation. All maps are a Mollweide projection and redrawn from Scotese (2021).

Starting in the late Albian a major marine transgression formed the Skull Creek Seaway (Fig. 11), which divided North America into the isolated landmasses of Laramidia and Appalachia, ending this period of cosmopolitanism and faunal exchange (Blakey & Ranney, 2018; Slattery et al., 2015). This marine transgression coincides with the depositional hiatus observed in the terrestrial record between the Trinity and Woodbine Groups, when Texas was largely inundated (Winkler et al., 1995). Sea level fell briefly during the earliest Cenomanian, re-establishing a connection between the landmasses that ran through the central United States (Scotese, 2021; Slattery et al., 2015). By the middle Cenomanian, the Greenhorn Transgression formed the Western Interior Seaway (WIS), with the Woodbine Group creating an extensive delta system along the southwestern flank of Appalachia (Blakey & Ranney, 2018; Scotese, 2021; Slattery et al., 2015).

By Cenomanian time a diverse array of theropod taxa appear in Laramidia including dromaeosaurine and velociraptorine dromaeosaurids, troodontids, *Richardoestesia*-like coelurosaurs, small tyrannosauroids such as *Moros intrepidus*, and the large carcharodontosaur *Siats meekerorum* in the Cedar Mountain (Mussentuchit Member) and Naturita (“Dakota”) Formations, demonstrating a major turnover in the terrestrial fauna was underway (Cifelli et al., 1999; Eaton et al., 1999; Zanno & Makovicky, 2013; Zanno et al., 2019).

The Lewisville theropod community is distinctly similar, including a large carcharodontosaur, mid-sized tyrannosauroid, ornithomimosaur, large dromaeosaurine, *Richardoestesia*-like coelurosaur, and troodontid. However, due to the history of two distinct highstand events in the late Albian and middle Cenomanian, two equally plausible scenarios may explain the similarity of the Lewisville assemblage to western deposits: (1) it was due to the initial division of a cosmopolitan fauna in the late Albian, or (2) it was due to dispersal between Laramidia and Appalachia during the early Cenomanian regression. Currently available data are unable to distinguish between these two hypotheses.

Currently no deposits contemporaneous to the Woodbine Group have produced theropod material elsewhere in Appalachia. The only exception is a single distal metatarsal assigned to a tyrannosauroid, possibly from the Raritan Facies of the Potomac Formation in New Jersey, however its exact provenance is uncertain (Baird, 1989; Brownstein, 2018a). Despite this lack of comparable material, the Lewisville Formation does show that ornithomimosaurs, dromaeosaurids, and tyrannosauroids were present in western Appalachia during or shortly after WIS formation. However, the evolutionary relationships between the Lewisville material and younger specimens from Appalachia at this time cannot be determined without more diagnostic remains.

### A transitional fauna

A growing record from the mid-Cretaceous provides a detailed record of Laramidian terrestrial ecosystems in transition. Formerly dominant groups of dinosaurs, crocodyliforms, mammals, and other vertebrates that characterized earlier Cretaceous communities are replaced by new groups in a millions-year long reorganization of terrestrial ecosystems (Kirkland et al., 1999; Nydam, 2013; Zanno & Makovicky, 2013).

The Lewisville Formation assemblage differs from the earlier Trinity Group assemblage, but maintains elements of the earlier cosmopolitan fauna, including a large-bodied carcharodontosaur, mid-sized tyrannosauroid, ornithomimosaur, and large dromaeosaurine, similar to Albian and Cenomanian deposits from the west. The presence of specimens in the Lewisville fauna similar to *Richardoestesia* and the troodontid *Paronychodon* from the Cedar Mountain Formation (Mussentuchit Member), both taxa associated with Campanian-Maastrichtian communities and unknown from North America before the Cenomanian, mark an early stage in the emergence of taxa common in later Late Cretaceous faunal assemblages. The mixture of these different theropod groups implies a gradual, rather than abrupt, ecological shift in dominant taxa from the Albian through the early Cenomanian of North America. Similar patterns are documented in Lewisville crocodyliforms, turtles, and fish, although the biogeographic patterns differ between groups, implying complex, clade-specific responses (Adams, Noto & Drumheller, 2017; Adrian et al., 2019; Adrian et al., 2021; Cavin et al., 2021). This complexity is due, at least in part, to the two separate marine transgressions in the late Albian and middle Cenomanian that severed the connection between Laramidia and Appalachia. In the case of the Lewisville Formation assemblage, the remnants of a formerly cosmopolitan fauna and potentially recent immigrants crossing from the west were isolated *via* Cenomanian marine high stands and became integrated into a distinctive local fauna on the southwestern coast of Appalachia. Low-latitude faunal assemblages in the mid-Cretaceous evolved in response to major faunal interchange, sea level, and climate change, creating unique communities that led to a major increase in taxonomic diversity throughout this transition and set the stage for the emergence of later Late Cretaceous faunal assemblages.

## Conclusions

The Lewisville Formation theropod assemblage adds new information to the poorly known mid-Cretaceous interval in North America. This new theropod material includes at least 6–7 taxa representing small, medium, and large theropod dinosaurs. Specimens representing the Carcharodontosauria, Tyrannosauroidea, and Troodontidae mark the first occurrence of each group in Appalachia. As the most fossiliferous and diverse terrestrial assemblage from the Cenomanian of Appalachia, the Lewisville Formation facilitates comparisons with Cretaceous-age units between eastern and western North America. Comparison with assemblages across North America supports the presence of a cosmopolitan fauna throughout the Early Cretaceous, when separate marine transgressions in the late Albian and early Cenomanian separated Laramidia and Appalachia, followed by a sustained evolutionary divergence that continued through much of the Late Cretaceous (Adams, Noto & Drumheller, 2017; Brownstein, 2018a; Noto et al., 2020). The theropod assemblage found in the Woodbine Group differs from older Trinity Group assemblages, and is remarkably similar to contemporaneous deposits in Laramidia to the west (Jacobs & Winkler, 1998; Kirkland et al., 1999; McDonald et al., 2017; Zanno & Makovicky, 2013; Zanno et al., 2019). The Lewisville Formation record indicates that the faunal transition between Early- and Late Cretaceous-dominant groups was already underway by early-middle Cenomanian time, which was gradual and biogeographically complex. Continuing research into the Woodbine Group of the Dallas-Fort Worth area is expected to shed more light on the paleoecology and paleobiogeography of Cenomanian communities and their role in understanding larger patterns of change occurring globally throughout this interval (Adrian et al., 2019; Adrian et al., 2021; Drumheller et al., 2021; Hacker & Shimada, 2021; Noto et al., 2020).

## Supplemental Information

10.7717/peerj.12782/supp-1 Supplemental Information 1 Graph of tibial midshaft robustness ratios for a selection of large theropod taxa. Midshaft height to tibial length ratio (AP:L) plotted as a function of midshaft width to tibial length ratio (ML:L). The two length estimates for the Lewisville Formation ornithomimosaur tibia are given as ’low’ (560 mm) and ’high’ (600 mm).

10.7717/peerj.12782/supp-2 Supplemental Information 2 Measurements of select large theropod tibiae. The total length for SMU 76806 is estimated between 560 and 600 mm, with both ends of the range used for comparison. * = estimated from published figure, † = estimated total length.

10.7717/peerj.12782/supp-3 Supplemental Information 3 Theropod taxa and tooth measurements used for PCA and discriminant function analyses, with results for both incomplete and reconstructed tooth datasets. The raw data show all tooth measurements and taxa used for each analysis, as well as the complete results of all analyses, including the incomplete and reconstructed tooth measurement datasets discussed in the text.

## Institutional abbreviations

BMRP: Burpee Museum of Natural History, Rockford, Illinois, USA
DMNH: Perot Museum of Nature and Science (formerly the Dallas Museum of Natural History), Dallas, Texas, USA
FMNH: Field Museum of Natural History, Chicago, Illinois, USA
SMU: Shuler Museum of Paleontology, Southern Methodist University, Dallas, Texas, USA.

## Additional Information and Declarations

### Data Availability

The following information was supplied regarding data availability: The raw measurements and used for the PCA and Discriminant Function analyses and their results are available in the Supplemental File.
