# Supplementary figures and images for: A newly recognized theropod assemblage from the Lewisville Formation (Woodbine Group; Cenomanian) and its implications for understanding Late Cretaceous Appalachian terrestrial ecosystems
Source: PeerJ. 2022 Jan 25;10:e12782. doi: 10.7717/peerj.12782 (PMC8796713; doi:10.7717/peerj.12782)

Figure S1

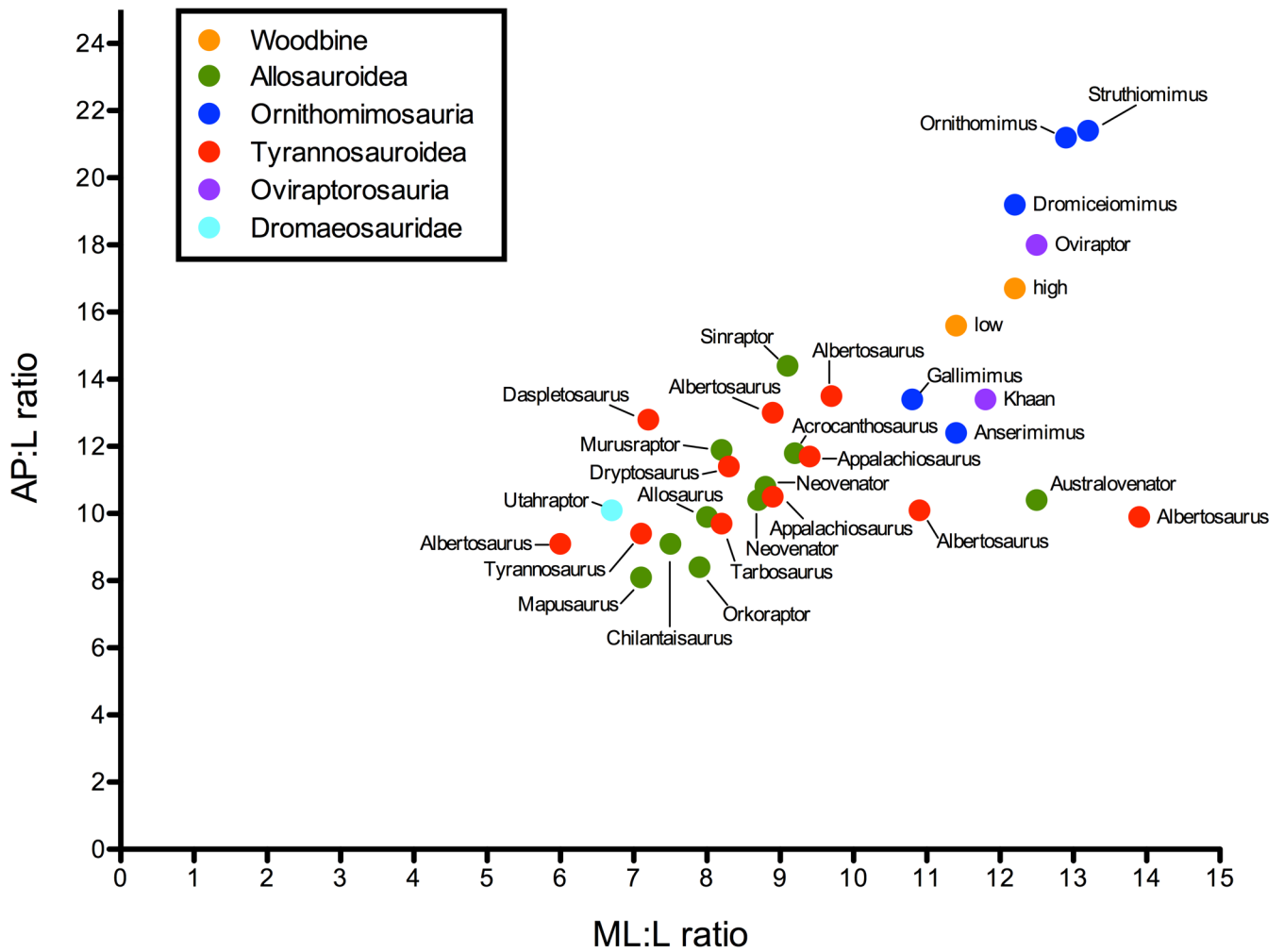

Supplement: Supplemental Information 1 — Midshaft height to tibial length ratio (AP:L) plotted as a function of midshaft width to tibial length ratio (ML:L). The two length estimates for the Lewisville Formation ornithomimosaur tibia are given as ’low’ (560 mm) and ’high’ (600 mm). [file peerj-10-12782-s001.pdf]
