# Supplementary material for: A newly recognized theropod assemblage from the Lewisville Formation (Woodbine Group; Cenomanian) and its implications for understanding Late Cretaceous Appalachian terrestrial ecosystems
Source: PeerJ. 2022 Jan 25;10:e12782. doi: 10.7717/peerj.12782 (PMC8796713; doi:10.7717/peerj.12782)
Supplement: Supplemental Information 2 — The total length for SMU 76806 is estimated between 560 and 600 mm, with both ends of the range used for comparison. * = estimated from published figure, † = estimated total length. [file peerj-10-12782-s002.docx]

Table S1: Measurements of select large theropod tibiae. The total length for SMU 76806 is estimated between 560 and 600 mm, with both ends of the range used for comparison.

| Group | Taxon | Length (L) | Midshaft width (ML) | Midshaft height (AP) | ML:L ratio | AP:L ratio | Reference |
| --- | --- | --- | --- | --- | --- | --- | --- |
| Woodbine | SMU 76809 low | 560† | 49 | 36 | 11.4 | 15.6 | This study |
| Woodbine | SMU 76809 high | 600† | 49 | 36 | 12.2 | 16.7 | This study |
| Allosauroid | Allosaurus | 747 | 93.5 | 75.5 | 8.0 | 9.9 | Christiansen and Farina 2004 |
| Allosauroid | Sinraptor | 820 | 90.5 | 57 | 9.1 | 14.4 | Christiansen and Farina 2004 |
| Neovenatoridae | Neovenator (right) | 685 | 79 | 66 | 8.7 | 10.4 | Brusatte et al., 2008 |
| Neovenatoridae | Neovenator (left) | 680 | 77 | 63 | 8.8 | 10.8 | Brusatte et al., 2008 |
| Neovenatoridae | Chilantaisaurus | 954 | 128* | 105* | 7.5 | 9.1 | Benson and Xing 2008 |
| Megaraptora | Australovenator (right) | 564 | 45 | 54 | 12.5 | 10.4 | White et al., 2013 |
| Megaraptora | Murusraptor | 690 | 84 | 58 | 8.2 | 11.9 | Coria and Currie 2016 |
| Megaraptora | Orkoraptor | 700* | 89 | 83* | 7.9 | 8.4 | Novas et al., 2008 |
| Carcharodontosauria | Acrocanthosaurus | 865 | 94 | 73 | 9.2 | 11.8 | Stovall and Langston 1950 |
| Carcharodontosauria | Mapusaurus | 1040* | 146* | 128* | 7.1 | 8.1 | Coria and Currie 2006 |
| Ornithomimosauria | Gallimimus | 737 | 68 | 55 | 10.8 | 13.4 | Christiansen and Farina 2004 |
| Ornithomimosauria | Anserimimus | 472 | 41.5 | 38 | 11.4 | 12.4 | Christiansen and Farina 2004 |
| Ornithomimosauria | Struthiomimus | 534 | 40.5 | 25 | 13.2 | 21.4 | Christiansen and Farina 2004 |
| Ornithomimosauria | Ornithomimus | 498 | 38.5 | 23.5 | 12.9 | 21.2 | Christiansen and Farina 2004 |
| Ornithomimosauria | Dromiceiomimus | 537 | 44 | 28 | 12.2 | 19.2 | Christiansen and Farina 2004 |
| Tyrannosauroidea | Appalachiosaurus (right) | 763.5 | 85.5 | 72.4 | 8.9 | 10.5 | Carr et al., 2005 |
| Tyrannosauroidea | Appalachiosaurus (left) | 780.7 | 83.1 | 67 | 9.4 | 11.7 | Carr et al., 2005 |
| Tyrannosauroidea | Dryptosaurus | 778 | 94.1 | 68.5 | 8.3 | 11.4 | Carr et al., 2005 |
| Tyrannosauroidea | Albertosaurus | 987 | 71 | 99.5 | 13.9 | 9.9 | Christiansen and Farina 2004 |
| Tyrannosauroidea | Albertosaurus ROM 1247 (right) | 793.7 | 81.9 | 58.8 | 9.7 | 13.5 | Carr et al., 2005 |
| Tyrannosauroidea | Albertosaurus ROM 1247 (left) | 790.7 | 72.3 | 78 | 10.9 | 10.1 | Carr et al., 2005 |
| Tyrannosauroidea | Albertosaurus CMN 350 | 842 | 140 | 93 | 6.0 | 9.1 | Carr et al., 2005 |
| Tyrannosauroidea | Albertosaurus ROM 807 | 998.3 | 112.6 | 76.9 | 8.9 | 13.0 | Carr et al., 2005 |
| Tyrannosauroidea | Tarbosaurus | 799 | 97 | 82 | 8.2 | 9.7 | Christiansen and Farina 2004 |
| Tyrannosauroidea | Daspletosaurus | 1017 | 141.5 | 79.5 | 7.2 | 12.8 | Christiansen and Farina 2004 |
| Tyrannosauroidea | Tyrannosaurus | 1199 | 170 | 128 | 7.1 | 9.4 | Christiansen and Farina 2004 |
| Oviraptorosauria | Oviraptor | 388 | 31 | 21.5 | 12.5 | 18.0 | Christiansen and Farina 2004 |
| Oviraptorosauria | Khaan | 224 | 19* | 16.7* | 11.8 | 13.4 | Balanoff and Norell 2012 |
| Dromaeosauridae | Utahraptor | 505 | 75* | 50* | 6.7 | 10.1 | Kirkland et al., 1993 |

* = estimated from published figure † = estimated total length
